# Bioconversion of Grape Pomace with *Rhizopus oryzae* under Solid-State Conditions: Changes in the Chemical Composition and Profile of Phenolic Compounds

**DOI:** 10.3390/microorganisms11040956

**Published:** 2023-04-06

**Authors:** Gordana Šelo, Mirela Planinić, Marina Tišma, Josipa Martinović, Gabriela Perković, Ana Bucić-Kojić

**Affiliations:** Faculty of Food Technology Osijek, Josip Juraj Strossmayer University of Osijek, F. Kuhača 18, HR-31 000 Osijek, Croatia

**Keywords:** grape pomace, solid-state fermentation, *Rhizopus oryzae*, chemical composition, phenolic compounds recovery

## Abstract

Grape pomace is a sustainable source of bioactive phenolic compounds used in various industries. The recovery of phenolic compounds could be improved by biological pretreatment of grape pomace, as they are released from the lignocellulose structure by the activity of the enzymes produced. The influence of grape pomace pretreatment with *Rhizopus oryzae* under solid-state conditions (SSF) on the phenolic profile and chemical composition changes was studied. SSF was performed in laboratory jars and in a tray bioreactor for 15 days. Biological pretreatment of grape pomace resulted in an increase in the content of 11 individual phenolic compounds (from 1.1 to 2.5-fold). During SSF, changes in the chemical composition of the grape pomace were observed, including a decrease in ash, protein, and sugar content, and an increase in fat, cellulose, and lignin content. A positive correlation (*r* > 0.9) was observed between lignolytic enzymes and the hydrolytic enzyme’s xylanase and stilbene content. Finally, after 15 days of SSF, a weight loss of GP of 17.6% was observed. The results indicate that SSF under experimental conditions is a sustainable bioprocess for the recovery of phenolic compounds and contributes to the zero-waste concept by reducing waste.

## 1. Introduction

Grape pomace (GP) is generally considered as waste, and its disposal is a major challenge for wineries. As a raw material, GP has been the subject of numerous studies, especially in the field of production of high-value products, such as polyphenols [1,2], organic acids [3], enzymes [4,5], GP extracts with improved antioxidant and prebiotic activity [6], application to fertilizer production [7], and others [8]. The high content of lignocellulosic components in GP requires the development of biorefinery approaches aimed at recycling GP and separating some high-value products, such as polyphenols. In order to break the complex lignocellulosic structure, release some bioactive compounds, and improve the nutritional properties of biomass, such as GP, different pretreatment methods (chemical, physical, biological) are used, with biological processes having advantages because they are efficient, cost-effective, and environmentally friendly [9,10]. Since the abovementioned properties are characteristic of solid-state fermentation (SSF), this biological treatment is often used as a pretreatment method for lignocellulosic biomass [5,10,11,12]. SSF involves different microorganisms that produce a complex enzyme system during growth that is responsible for breaking down complex molecules into simpler ones and releasing them from the lignocellulosic structure [11]. Moreover, SSF is a sustainable process that has the potential to recover bioactive phenolic compounds and valorize waste materials, such as pineapple waste, wheat bran, brewer’s grains, etc. [5,11,12]. Valorized material with enhanced nutritional properties has the potential to be used as animal feed [10,13]. Although bacteria, yeasts, and fungi can be used in SSF processes, filamentous fungi are most commonly used due to their ability to grow on complex solid substrates and produce a wide range of extracellular enzymes [14,15]. Microorganisms used in SSF are generally regarded as safe (GRAS), suggesting that products derived from SSF are largely safe for humans and animals [16]. One of these microorganisms is *Rhizopus oryzae*, which has the potential to produce various components of industrial interest, such as lactic acid, fumaric acid, enzymes (lipase, glucoamylase, polygalacturonase), and others [15,17,18]. It is known for its use in the production of traditional oriental foods [15]. *R. oryzae* is widespread in nature and is known as a primary or secondary colonizer due to its rapid growth and invasion of substrates rich in simple sugars (digestible substrates). *R. oryzae* belongs to the phylum *Zygomycota* and is characterized by sexual reproduction via zygospores, asexual reproduction through the formation of sporangiospores, and in most species, by non-septate hyphae. One of the main characteristics of the genus *Rhizopus* is the formation of rhizoids [15].

In this work, SSF was used for the bioconversion of GP by *R. oryzae*, first at a smaller laboratory scale in jars and then the process was carried out at a larger laboratory scale in a tray bioreactor. The aim was to investigate the influence of SSF on the changes in chemical composition and on the recovery of phenolic compounds from GP. Chemical compositions with an emphasis on phenolic compounds, as well as biomass concentration, content of minerals, heavy metals, polyaromatic hydrocarbons (PAHs), polychlorinated biphenyls (PCBs), and mycotoxins were analyzed and the activities of hydrolytic and lignolytic enzymes were measured.

## 2. Materials and Methods

### 2.1. Standards and Reagents

In this study, the following standards and reagents were used: analytical grade ethanol, potassium hydroxide, sodium chloride, and ascorbic acid that were acquired from Gram Mol Ltd. (Zagreb, Croatia), ultra-gradient grade methanol from J.T. Baker (Arnhem, The Netherlands), HPLC grade acetonitrile from Fisher Chemical (Loughborough, UK), and glacial acetic acid from Macron Fine Chemicals (Gliwice, Poland). Ergosterol, gallocatechin gallate, epicatechin gallate, (−)-epicatechin, (+)-catechin hydrate, caffeic acid, ellagic acid, gallic acid, resveratrol, syringic acid, kaempferol, *o*-coumaric acid, *p*-coumaric acid, ferulic acid, rutin hydrate, and *p*-hydroxybenzoic acid were purchased from Sigma Aldrich (Saint Louis, MO, USA). Procyanidin B1 and procyanidin B2 were obtained from Extrasynthese (Genay, France). Quercetin, 3,4-dihydroxybenzoic acid, and vanillic acid were obtained from Acros Organics (Geel, Belgium). ε-Viniferin was obtained from AppliChem (Darmstadt, Germany). Tyrosol, epigallocatechin, chlorogenic acid, sinapic acid, *p*-hydroxyphenylacetic acid, myricetin, Tween-80, DL-arabinose, maltotriose, L-rhamnose, sodium sulfite, *n*-octanol, 2,2-diphenyl-1-picrylhydrazyl (DPPH), 2,4,6-tris(2-pyridyl)-s-triazine (TPTZ), and 2,2’-azino-bis(3-ethylbenzothiazoline-6-sulfonic acid) diammonium salt (ABTS) were obtained from Sigma Aldrich (Saint Louis, MO, USA). Cellulose, D(+)-xylose, and iron(II)sulfate heptahydrate were obtained from Kemika (Zagreb, Croatia). D(−)-fructose, D(−)-ribose, D(+)-cellobiose, D(+)-galactose, D(+)-maltose monohydrate, D(+)-mannose, D(+)-sucrose, and copper sulfate were obtained from Acros Organics (Geel, Belgium). D(+)-glucose was obtained from Gram Mol Ltd. (Zagreb, Croatia). Potassium hydrogen phthalate, Folin–Ciocalteu reagent, and sodium carbonate anhydrous were acquired from Kemika (Zagreb, Croatia). Aluminum chloride hexahydrate was acquired from Alfa Aesar GmbH & Co KG (Kandel, Germany), and sodium nitrite, sodium sulfate, sodium hydroxide, and acetone from Gram Mol Ltd. (Zagreb, Croatia). Hydrochloric acid, sulfuric acid, n-butanol, and n-hexane were obtained from Carlo Erba Reagents GmbH (Emmendingen, Germany) and water was deionized in a Milli-Q water purification system (Millipore, Bedford, MA, USA).

### 2.2. Substrate and Microorganism

The initial sample of GP variety Frankovka (harvest 2017) was provided by a local winery (Feravino, Feričanci) in eastern Croatia and consisted of skin, pulp, seeds, and stems. After collection, GP was stored at −20 °C before being used as substrate in SSF.

The biological treatment of GP was performed by filamentous fungus *Rhizopus oryzae* (Faculty of Pharmacy and Biochemistry, Zagreb, Croatia) cultivated on a potato dextrose agar (PDA) medium for three days at 27 °C.

### 2.3. Biological Treatment of Grape Pomace by Rhizopus oryzae

Fifty grams of defrozen and coarsely crushed GP using a blender (Philips, HR 2860, Zagreb, Croatia) was mixed with 30 mL of distilled water in 720 mL laboratory jars and was autoclaved (121 °C/15 min) and cooled to room temperature. GP was inoculated with a spore suspension, which was prepared by resuspending the spores formed on PDA in Petri dishes in a Tween-80 solution (0.1% *v*/*v*). The spore concentration in the prepared suspensions was 1 × 10^7^ spores/mL. The moisture content of the substrate after inoculation was about 70%. Incubation was performed at 27 °C in an incubator with a fan set to 10% (KB 115, BINDER GmbH, Tuttlingen, Germany) for 15 days. The height of the substrate layer in the jars was 4–4.5 cm. The control sample of GP was prepared in the same way, with the difference being that the same amount of sterile water was added instead of the spore suspension, corresponding to day “0”.

SSF in the tray bioreactor was performed in such a way that after sterilization of the bioreactor and reaching the desired temperature in the bioreactor (27 °C), pre-sterilized GP (*m* = 1150 g) mixed with 150 mL of water was distributed on plates and cooled to 27 °C. Inoculation of the substrate on the plates was performed by adding 25% of the inoculum in proportion to the total mass of substrate on the plate, slightly mixing the inoculum with the substrate. The inoculum was prepared by cultivating *R. oryzae* in laboratory jars for 5 days according to the protocol described above. The moisture content of the substrate after inoculation was about 70%, and the height of the substrate layer on the plate was 2 cm. The control GP sample (day “0”) was prepared with sterile water instead of spore suspension. The same procedure was performed in SSF in laboratory jars. The process took place under natural aeration without mixing, and humidification in the bioreactor was via an external container of sterile water. SSF in the tray bioreactor was performed for 15 days.

After biological treatment in jars and tray bioreactor samples were sterilized (121 °C/15 min), the samples were dried at room temperature for 48 h and ground to a particle size of ≤1 mm using an ultracentrifugal mill (Retsch ZM200, Haan, Germany) to complete the SSF process. Samples prepared in this way were stored at +4 °C until extraction and further analysis.

### 2.4. pH Measurement

Two grams of the biologically treated GP were suspended in 10 mL of distilled water, vortexed for 30 min, centrifuged at 10,000× *g*, and the supernatant was used to measure pH with a pH meter (HI 2211 pH/ORP Meter, Hanna instruments, Zagreb, Croatia).

### 2.5. Chemical Composition of Grape Pomace

The results of chemical composition analysis refer to the dry mass of the sample, and all measurements were performed in triplicate. Results were reported as the mean value of replicates ± standard deviation (SD).

Solid GP samples before and after biological treatment (particle size ≤ 1 mm) were used for analysis of dry matter content; ash content according to AACC-08-03 method [19]; neutral detergent fiber (NDF) content, acid detergent fiber (ADF) content, and acid detergent lignin (ADL) content according to the Van Soest method [20]; crude protein content by the Kjeldahl method [21]; total carbon (TC) and inorganic carbon (IC) content, from which the percentage of total organic carbon was calculated (TOC_GP_ = TC − IC); free fat content according to the Soxhlet method [22]; and biomass concentration according to method of Barreira et al. [23].

The liquid extracts were prepared from solid GP samples for analysis of total phenolic compounds (TPC) by the Folin–Ciocalteu colorimetric method [24]; total flavonoids (TF) [25]; total extractable proanthocyanidins (TPA) [26]; phenolic compounds profile [27]; and antioxidant activity. Briefly, 1 g of solid GP was extracted with 40 mL of ethanol: water (1:1) in stoppered flasks at 80 °C. Extractions were performed in a water bath (Julabo, SW-23, Seelbach, Germany) by shaking at 200 rpm for 120 min in triplicate. After extraction, samples were centrifuged at 10,000× *g* for 10 min (Z 326 K, Hermle Labortechnik GmbH, Wehingen, Germany) and supernatant was used for the determination of phenolic compounds’ content.

To measure total nitrogen content (TN), total organic carbon (TOC_E_), reducing sugar, and individual sugar concentration [28], the extracts were prepared by extraction of 1 g of solid GP with 25 mL of distilled water in sealed flasks. The extraction was performed in a water bath by shaking at 170 rpm for 30 min at 30 °C in three replicates. After extraction, the suspension was centrifuged at 10,000× *g* for 10 min and supernatant was used for analysis.

Apart from the above analyzes, which are described in detail in the article published by Šelo et al. [28], the remaining analyzes are described in the following sections.

#### 2.5.1. Reducing Sugars

The concentration of reducing sugars was spectrophotometrically determined by the DNS method [29,30]. Briefly, 500 μL of extract GP and 500 μL of DNS reagent were added to a test tube and mixed in vortex, then the mixture was incubated at 100 °C for 5 min. The mixture was then cooled to room temperature and absorbance was measured at 540 nm. The blank was prepared in the same way, except that distilled water was used instead of extract.

#### 2.5.2. Biomass Concentration

Biomass concentration was determined using an indirect method by determining ergosterol concentration following the protocol of Barreira et al. [23] with some modifications. Saponification was performed by adding 2 mL ascorbic acid (0.1 M) and 10 mL potassium hydroxide (2 M) to the flasks containing the extract obtained after the analysis of free fats in the solid samples of GP before and after biological treatment. The flasks were shaken for 45 min at 60 °C in a water bath with a shaker at 125 rpm, then the contents of the flasks were cooled to room temperature and filtered into test tubes, followed by extraction with n-hexane. The n-hexane fractions were collected in test tubes where sodium sulfate was added to the tip of the spatula to dry the residual water. The contents of the tube were mixed on a vortex, filtered into an evaporation flask, and evaporated on a rotavapor (Büchi B-210, Flawil, Switzerland) at 40 °C. After evaporation, the contents of the flask were dissolved by adding 2 mL of methanol, which was filtered through a membrane with a pore size of 0.45 µm (Chromafil Xtra PTFE) and was used for UHPLC analysis.

UHPLC (UHPLC Nexera XR, Shimadzu, Kyoto, Japan) analysis of ergosterol concentration was performed by PDA detection by recording the spectrum at 280 nm and separation using a Shim-Pack GIST C18 column (250 × 4.6 mm, 3 μm, Shimadzu, Kyoto, Japan) and applying the isocratic method with methanol as the mobile phase at a flow rate of 1 mL/min at 25 °C for 20 min. The sample injection volume was 20 µL. Ergosterol in the samples was identified by comparing the retention time and UV–Vis spectrum with an authentic standard analyzed under the same chromatographic conditions. Ergosterol concentration was expressed as mg of ergosterol per gram of fats (mg/g_F_).

#### 2.5.3. Analysis of Minerals and Undesirable Substances

Analysis of minerals, heavy metals, and mycotoxins was performed in an accredited laboratory. Phosphorus content was spectrophotometrically analyzed according to the HRN ISO 6491:2001 method [31]. The content of calcium, potassium, iron, lead, cadmium, and arsenic was analyzed by method RU-305-05 at ICP-MS, which is an accredited method in the flexible area according to the requirements of the standard HRN EN ISO/IEC 17025:2017 [32]. Polyaromatic hydrocarbons (PAHs) and polychlorinated biphenyls (PCBs) were analyzed by methods RU-256-02 and RU-256-05 at GC-MS. Mycotoxins were analyzed by method RU-287-04 using LC-MS/MS (HRN EN ISO/IEC 17025:2017) [32].

### 2.6. Determination of Antioxidant Activity

The antioxidant activity (AA) of the extracts of GP, which were biologically treated with *R. oryzae* for 15 days, was determined by the DPPH, FRAP, and ABTS methods using a UV–VIS spectrophotometer (UV/VIS Spectrophotometer UV-1280, Shimadzu, Kyoto, Japan).

The DPPH assay was performed according to the method of Bucić-Kojić et al. [33]. Briefly, 3.9 mL of ethanol solution of DPPH radical (0.026 mg_DPPH_/mL) was added to 0.1 mL of extract prepared according to Section 2.5, and the absorbance of the reaction mixture was measured after 30 min of incubation in the dark at 515 nm. Absolute ethanol was used as a blank.

The ABTS assay was performed according to the method of Re et al. [34], with some modifications. Briefly, 950 µL of a diluted ABTS^•+^ radical solution was added to 50 µL of the extracts. The absorbance was measured after 10 min of incubation in the dark at 734 nm. The control sample was prepared in the same way, but ethanol was used instead of the sample. Absolute ethanol was used as a blank.

The FRAP assay was performed according to the method of Benzie and Strain [35], with some modifications. Prior to analysis, the reagent FRAP was prepared from 25 mL of 300 mM acetate buffer (pH 3.6) heated to 37 °C, 2.5 mL of 10 mM TPTZ solution (dissolved in 40 mM HCl), and 2.5 mL of 20 mM FeCl_3_(H_2_O)_6_ solution. Samples for analysis were prepared by mixing 2.7 mL of FRAP reagent, 270 μL of distilled water, and 150 μL of GPE, and the absorbance was read at 592 nm after 40 min of incubation in the dark at 37 °C. The blank sample was prepared in the same way, but distilled water was used instead of the extract.

All assays were performed in triplicate, and results were expressed in Trolox equivalents per dry basis of GP (g_TROLOX_/g_db_).

### 2.7. Measurements of Enzyme Activities

#### 2.7.1. Preparation of Crude Enzyme Extracts

After 1, 5, 10, and 15 days of SSF, 2 g of the homogenized GP sample was extracted in 10 mL of the buffer appropriate for each enzyme, as listed in Table 1. Extraction was performed with a vortex every 5 min at 15 s intervals (30 min total) followed by centrifugation at 10,000× *g* for 5 min. The supernatant was used to measure enzyme activity according to the assays described in Section 2.7.2.

#### 2.7.2. Assays for Measuring Enzyme Activity

Enzyme activities of hydrolytic enzymes (xylanase, cellulase, *β*-glucosidase, and invertase) and lignolytic enzymes (laccase, manganese peroxidase, and lignin peroxidase) were measured in triplicate using a UV–VIS spectrophotometer.

The activities of the hydrolytic enzymes xylanase (endo-1,4-β-xylanase) and cellulase (endoglucanases and exoglucanases) were determined by DNS method [36,37,38], while *β*-glucosidase activity was determined according to the study of Karpe et al. [39], and invertase activity was determined according to the method of Margetić and Vujčić [40].

The activities of the lignolytic enzymes MnP and laccase were determined by monitoring oxidation of the substrate 2,6-dimethoxyphenol (DMP) at 469 nm for 120 s according to the method of Lueangjaroenkit et al. [41]. The assay for LiP was performed according to the method described by Field et al. [42] and Linko and Haapala [43] by monitoring the oxidation of the substrate veratryl alcohol at 310 nm.

### 2.8. Statistical Analysis

Student’s *t*-test at 95% significance level (*p* < 0.05) by TIBCO Statistica software (TIBCO Software Inc., Palo Alto, CA, USA) was used to compare the mean values of individual phenolic contents between the “0” day (untreated sample) and the day of fermentation when the maximum yield of phenolic compounds was obtained. To test the significance of the difference between the arithmetic means of the samples representing populations, one-way analysis of variance (ANOVA) was performed. After ANOVA showed the presence of statistically significant differences between the arithmetic means of the observed populations, further analysis was conducted using a post-hoc test, i.e., Duncan’s test for multiple ranges, to determine between which populations with arithmetic means there was a significant difference (*p* < 0.05). Samples belonging to the same population are marked with the same lowercase letter of the alphabet in the figures/tables.

## 3. Results and Discussion

The cultivation of *R. oryzae* on GP was carried out under SSF conditions for 15 days in jars and a tray bioreactor, and the obtained results were presented in comparison. Based on the screening of the profile of individual phenolic compounds after biological treatment of GP with 11 microorganisms published in our previous work [28], *R. oryzae* proved to be one of the most potentially effective microorganisms for the recovery of most of the identified phenolic compounds from the Cabernet Sauvignon GP variety. Therefore, zigomycete *R. oryzae* was selected for cultivation on the Frankovka GP variety for 15 days.

### 3.1. Biological Treatment of Grape Pomace by R. oryzae in a Tray Bioreactor

**Figure 1:**
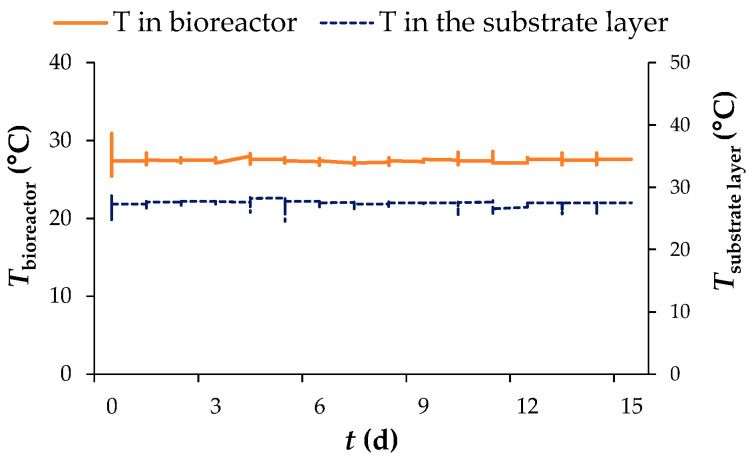
Temperatures in a tray bioreactor and in the substrate layer during SSF.

During the SSF process in a tray bioreactor, the temperature inside the bioreactor and inside the substrate layer was measured with temperature probes. The temperature in the bioreactor ranged from 26.7 °C to 28.0 °C and ranged from 26.8 °C to 28.3 °C in the middle of the substrate layer. As can be seen in Figure 1, the temperature was uniform during the SSF process, i.e., there were no significant temperature fluctuations, except for periodic sampling at 24 h intervals, which caused a temporary temperature drop of up to 25.4 °C in the bioreactor and up to 25.6 °C in the middle of the substrate layer.

### 3.2. Chemical Composition of Grape Pomace

The chemical composition of the initial GP sample, which is used as substrate in SSF, has a significant impact on the overall SSF process. The amount of polysaccharides and other components contained in the substrate strongly influences the growth of the microorganisms used and consequently, the production of enzymes during SSF. The growth and metabolism of the microorganisms ultimately affect the extractability of phenolic compounds from GP.

Before (day “0”) and after biological treatment (day 5, 10, and 15) of GP in laboratory jars and a tray bioreactor, the chemical composition was analyzed to investigate the effects of SSF by *R. oryzae* on the changes in chemical composition and the recovery of phenolic compounds from GP. The results of the chemical composition analysis are shown in Table 2. The GP before and after SSF were directly used for the determination of dry matter content, which was about 88–95%.

#### 3.2.1. Lignocellulosic Components

Lignin, cellulose, and hemicellulose content were calculated using NDF, ADF, and ADL, where hemicellulose content was calculated as the difference between NDF and ADF, and cellulose content was calculated as the difference between ADF and ADL, while ADL represents lignin content. After SSF of GP by *R. oryzae*, an increase in cellulose and lignin content was observed in laboratory jars and the tray bioreactor (Table 2).

Depending on the substrate, the proportion and composition of lignin may vary, leading to different effects of filamentous fungi on the degradation of lignocellulosic biomass. Moreover, each type of filamentous fungi exhibits different selectivity in the degradation of lignin [44]. According to the literature, the lignin content in GP varies from 11.6% to 41.3%, depending on the grape variety [45]. Similar results in studies on GP were published by Filippi et al. [3], where the lignin content was 34.79%, and by Teles et al. [46], where the lignin content was 40.24%. To better understand the process of biological treatment and to see which fractions are actually degraded, it is important to present the data as absolute values expressed in terms of the dry matter of the sample. In their study, Van Kuijk et al. [44] observed an increase in cellulose content after biological treatment of wheat straw, *Mischantus giganteus,* and sawdust with *Lentinula edodes,* and indicated that the absolute cellulose content cannot be increased during biological treatment with fungi, which they confirmed by presenting the results as absolute values considering the loss of dry matter after biological treatment. The reason for the increase in lignin and cellulose content could be the degradation of other components of the substrate when the fungus first consumes a large part of the readily available components [44,47]. Due to its high lignin content, GP is a difficult material to degrade. The characteristics that make lignocellulosic biomass resistant to decomposition are the crystalline structure of the cellulose, the hemicellulose being located between macro- and micro-fibril cellulose, and the lignin, which is responsible for structural stability and fills the space between the hemicellulose and covers the cellulose skeleton, creating lignocellulosic matrices [9]. The presence of lignin makes it difficult to access cellulose and hemicellulose. Therefore, lignocellulosic biomass must be pretreated for further use and for obtaining desired products, e.g., by converting cellulose and hemicellulose into simple sugars and fermenting them to produce biofuels or to produce other high-value products, including bioactive phenolic compounds [46,48]. Table 2 shows that hemicellulose content decreased after biological treatment of GP by *R. oryzae* in laboratory jars compared with the content in the biologically untreated sample (day “0”), which may be a consequence of the action of hydrolytic enzymes, such as xylanases, acting on the decomposition of xylan, the main unit of hemicellulose. However, in the tray bioreactor, hemicellulose content slightly decreased after the fifth day of fermentation and then increased until the 15th day of SSF, when a higher hemicellulose content was observed than in the biologically untreated sample.

#### 3.2.2. Sugars Content

By analyzing the individual sugars in the extracts obtained from GP before and after SSF with *R. oryzae*, sucrose, glucose, and fructose were quantified from a total of 13 analyzed individual sugar standards listed in Section 2.1. The amount of sucrose, glucose, and fructose decreased with the duration of SSF by *R. oryzae*, as did the amount of reducing sugars in the extracts of GP. Since filamentous fungi use monosaccharides and disaccharides from the substrate as a source of energy for their growth and development, and Frankovka GP is rich in sucrose, glucose, and fructose, it is assumed that the consumption of the mentioned sugars by the fungus is the reason for the decrease in their content during SSF. Moreover, the nutrients from the substrate were available to the filamentous fungus *R. oryzae* for its growth, but it did not reach the stage where (in the absence of nutrients) it would have had to break down complex molecules into simpler ones to obtain nutrients needed for its growth, which could be related to the fact that the content of complex components (lignin and cellulose) did not decrease during both processes. Therefore, in order to achieve better degradation of complex molecules and release of simple sugars from the lignocellulose structure, as well as better production of secondary metabolites, it is necessary to extend the duration of the SSF process.

#### 3.2.3. Ash, Proteins, Total Organic Carbon, Total Nitrogen, and Free Fats Content

As can be seen in Table 2, the ash content also decreased after SSF, by 12.3% in the laboratory jars and by 8.1% in the tray bioreactor after 15 days of fermentation, which according to Mushollaeni and Tantalum [49], may be an indicator that the microorganism uses some minerals, such as Fe, Na, Mg, Zn, and K, for its growth during fermentation. This consideration can also be applied to the present study, since after 15 days of SSF, a decrease in the content of certain elements (P, K, Fe) was observed in GP, which is shown in Section 3.2.6. (Table 3).

Crude protein content decreased in both processes until the 10th day of SSF, while an increase was observed on the 15th day. However, the value was still lower than the protein content in the initial biologically untreated sample (day “0”) during SSF in laboratory jars, while the protein content after treatment in the bioreactor reached the initial value after 15 days of SSF. Table 2 also shows a decrease in TN during fermentation, which may also be related to the metabolism of the microorganism. GP contains polymeric carbon sources, such as polysaccharides, and polymeric nitrogen sources, such as proteins. However, their utilization requires the presence of hydrolytic enzymes. Microorganisms primarily set out to utilize the readily metabolizable nitrogen and carbon available at a given time and suppress the utilization of other sources until the primary sources are depleted. In this way, they block the synthesis of enzymes involved in the degradation of other carbon and energy sources [50].

The TOC results presented in Table 2 show the consumption of organic carbon by microorganisms during the SSF process. In addition, carbon is continuously consumed and released during the SSF process, as shown by TOC results from analysis of the solid samples after SSF, reported as TOC_GP_ in Table 2. Analysis of the solid samples of GP before and after SSF did not detect inorganic carbon (IC), so TOC is equal to total carbon (TC), since the TOC value is calculated from the difference between TC and IC. During the SSF process, carbon is released, for example, in the form of glucose by the action of hydrolytic enzymes, especially cellulases [51,52]. Considering the availability of sugars in the substrate, e.g., sucrose, which is one of the main carbon sources (12.04 ± 0.27 mg/g_db_, day ”0”), and considering the high TOC_E_ values (79.25 ± 7.15 mg/g_db_, day “0”), which follow the trend of decreasing reducing sugars until the end of fermentation, it can be assumed that the sugars present in the initial sample of GP were the main carbon source for microbial growth and that the microorganism did not need to degrade a complex lignolytic structure to access the carbon source. It can also be assumed that more significant degradation of lignolytic components would occur if the duration of SSF was extended [10].

As for TN, Table 2 shows that the content in the biologically untreated sample was 1.45 ± 0.00 mg/g_db_ and that it continuously decreased during SSF in both processes, reaching a value of 28.3% of the initial content after the 15th day of fermentation in laboratory jars, i.e., 24.8% of the initial content during fermentation in the larger scale (tray bioreactor). These data are consistent with the data on protein content during the 10-day biological treatment, which was due to the action of enzymes involved in the degradation of proteins to provide the microorganism with the nitrogen necessary for growth and the performance of other metabolic processes. The increase in protein content after day 15 of SSF in the tray bioreactor compared with day 10 is due to the development of mycelia associated with an increase in ergosterol concentration in the biomass, as shown by the results in Section 3.2.5.

During the 15-day SSF of GP with *R. oryzae* in laboratory jars, a 43.6% increase in fat content was observed. In the process carried out in a tray bioreactor, the highest increase in fat content (48.4%) was observed after the 5th day of fermentation and then decreased until the end of fermentation. The accumulation of lipids in the culture medium is stimulated by restricted nutrient conditions, such as nitrogen limitation and excess carbon sources. In the initial phase of fermentation, when all required nutrients are present in high concentrations, the production of biomass with a lower lipid content and the consumption of C and N sources dominate. However, after a significant reduction in the nitrogen content of the fermentation medium, certain microorganisms began to accumulate lipids [53,54], which was the case in this process of biological treatment of GP with *R. oryzae* in laboratory jars. When the content of lipids begins to decrease, as was the case with SSF in a tray bioreactor after 10 and 15 days, this indicates that the microorganism is metabolizing them to provide energy for its growth [55].

#### 3.2.4. Loss of the Total Substrate Mass

**Figure 2:**
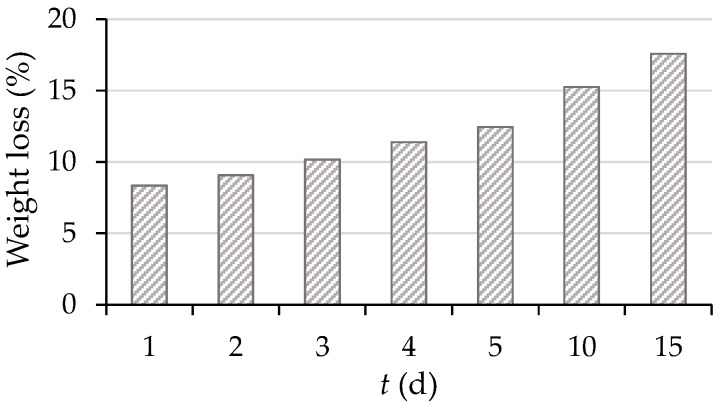
Substrate mass loss during SSF (1–5, 10, 15 day) by *R. oryzae* in laboratory jars.

Numerous parameters, such as the particle size of the substrate, moisture content, air flow, and temperature, affect the growth of microorganisms during the SSF process. The composition of the nutrient medium, i.e., the substrate used in SSF, is of great importance for the growth of microorganisms, but also for the production of secondary metabolites. During the SSF process, microorganisms use the nutrients from the substrate for their growth and development, and they produce a complex system of enzymes that catalyze biotransformation processes and thus participate in the degradation of the solid matrix of the substrate. This leads to the release of high-value matrix-bound components, such as bioactive compounds, and at the same time, to a loss of total substrate mass. These characteristics make SSF a potential technology for the production of high-value products and ultimately for reducing the amount of production residues [56]. Biological treatment of GP by *R. oryzae* for 15 days in laboratory jars resulted in a substrate mass loss of 17.6% when comparing the final substrate mass to the initial substrate mass (Figure 2).

#### 3.2.5. Biomass Concentration

*R. oryzae* is characterized by rapid growth and the ability to utilize various compounds and polysaccharides as sources of energy and carbon. Due to its rapid growth, it is known as a primary or secondary colonizer, with the ability to rapidly invade digestible substrates, i.e., substrates rich in simple sugars [15]. Since the separation of microbial biomass and substrate is not possible in SSF processes, it is almost impossible to determine the exact biomass concentration, and various indirect methods are used to evaluate it, such as determination of protein content, nucleic acids content, glucosamine, and ergosterol [57]. Determination of the ergosterol content, the primary sterol in the cell membranes of filamentous fungi, was used in this work as an indirect method to evaluate the growth of the microorganism used. Obtained results are expressed as per gram of fats (mg/g_F_).

**Figure 3:**
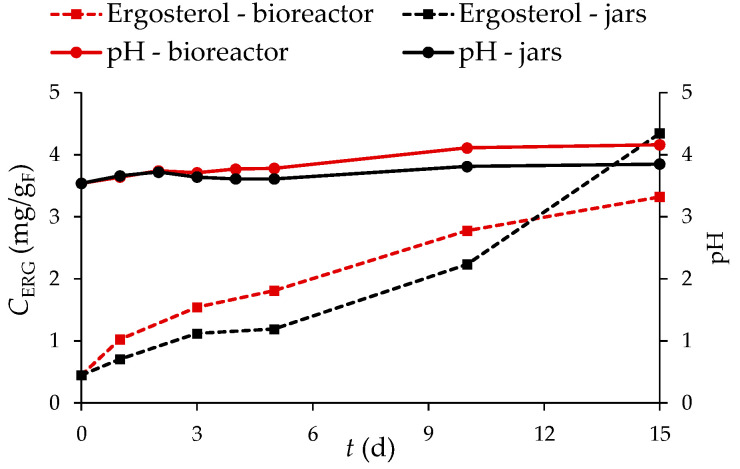
Ergosterol concentration (*C*_ERG_) and pH of extracts obtained from GP before (day “0”) and during the 15-day SSF process by *R. oryzae* in laboratory jars and tray bioreactor.

The highest ergosterol content was measured after 15 days of SSF of GP by *R. oryzae* in laboratory jars (4.34 mg/g_F_) and in the tray bioreactor (3.32 mg/g_F_). As can be seen in Figure 3, the ergosterol content in GP increases in both processes until day 15. The first five days of fermentation are characterized by an adaptation phase (lag phase), as the microorganisms need some time to adapt to the new environment. This is followed by exponential growth until day 15 of fermentation, indicating a phase of accelerated growth of the microorganism (exponential phase). The conditions under which the microorganism grows (temperature and availability of nutrients) have a great influence on the growth rate of the microorganism. In the process performed in the tray bioreactor, higher ergosterol content was observed from the beginning of fermentation, which was due to the fact that the substrate on the plate was inoculated with a culture already grown in laboratory jars. Adaptation and growth of *R. oryzae* was faster in the laboratory jars, where the inoculum was a spore suspension and the culture needed more time to adapt to the conditions and begin growth.

Figure 3 also shows that ergosterol content in the jars increased more after 10 days of fermentation than in the bioreactor. This is due to the increased production of microbial biomass during the fermentation process. Readily available sugars, e.g., glucose, were better utilized by the microorganism. This is reflected in a decrease in glucose content to 0.78 ± 0.69 mg/g_db_ (by 63.4% compared with the 10th day of SSF) in the laboratory jars and to 2.14 ± 0.22 mg/g_db_ (by 18.9% compared with the 10th day of SSF) in the bioreactor on the 15th day of fermentation. Looking at the results of sugar analysis during SSF (Table 2), it is clear that sugar degradation follows the trend of increasing biomass concentration.

Figure 3 also shows the pH of extracts obtained from GP before (day “0”) and after SSF (days 1–5, 10, and 15) with *R. oryzae*, which ranged from 3.54 to 3.85 in the laboratory jars and from 3.54 to 4.16 in the tray bioreactor. In Figure 3, a decrease in pH was observed on the third day of fermentation compared with the first two days, probably due to the production of organic acids. The increase in pH may be a consequence of the assimilation of organic acids by the microorganism [58], and it may also be associated with an increase in the activity of the protease enzyme, whose action leads to the release of amino acids from degraded proteins [59].

**Figure 4:**
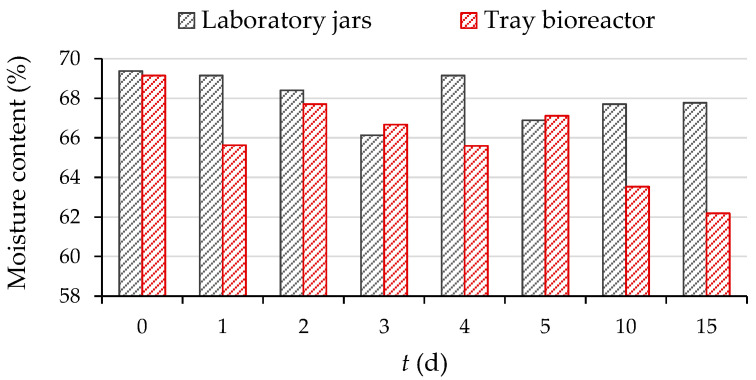
Moisture content of biomass during (day 0–5, 10, 15) SSF of GP by *R. oryzae* in laboratory jars and tray bioreactor.

The water present also plays an important role in the growth of microorganisms in SSF, due to its absorption in the substrate and due to the consumption of nutrients, for which dissolution and transport water is essential. As can be seen in Figure 4, the moisture content of the biomass gradually decreases during SSF in both processes. This is due to the evaporation of water, which was stimulated by the generation of metabolic heat that simultaneously compensated for the consumption of heat required for evaporation (latent heat of evaporation), and there was no temperature change in the biomass layer (Figure 1). The moisture content of the substrate decreased by 2.3% and 10.1% after 15 days of SSF in the laboratory jars and tray bioreactor, respectively. Natural aeration in a tray bioreactor prevented the spread of spores due to the rapid growth of *R. oryzae*, while humidification of the substrate on the plates by an external tank of sterile water prevented excessive drying of the biomass and kept the moisture content in the optimal range for microorganism growth and successful performance of the SSF process.

#### 3.2.6. Minerals and Undesirable Substances in Grape Pomace

The contents of minerals (P, Ca, K, Fe), heavy metals (Pb, Cd, As), and undesirable substances, such as PAHs and PCBs, as well as mycotoxins in the solid GP samples (before and after SSF) were analyzed to investigate the potential uses of GP. The results showed that Ca content increased during fermentation, while the content of the other minerals decreased (Table 3). The results also show that the content of Pb, Cd, and As (Table 3) in GP is within the permissible concentration limits. Additionally, undesirable substances, such as PAHs and PCBs, and the mycotoxins (Table 4) do not exceed the permissible concentrations in animal feed and GP can potentially be used as an additive in animal feed production.

The preparation of animal feed requires the presence of certain minerals necessary for animal health and productivity, and numerous trace elements are added to animal feed as additives. Some elements, such as As, Cd, F, Pb, and Hg, are considered undesirable if they exceed the prescribed permissible concentrations [5]. Table 3 shows the results of minerals and heavy metals detected in GP before and after 15 days of SSF by *R. oryzae*.

According to EU DIRECTIVE 2002/32/ EC [60], the maximum permissible concentration of arsenic in feed and complementary feed is 2 mg/kg, and for lead, 10 mg/kg, while the permissible concentrations for cadmium in feed materials of plant origin and in complementary feed are 1 and 0.5 mg/kg, respectively.

The total content of PAHs, PCBs, and mycotoxins in untreated and biologically treated GP is expressed as Limit of Quantification (LOQ); results are shown in Table 4.

PCBs in feed are a group of complex substances that can affect the hormonal, nervous, and immune systems of animals. The total PCBs LOQ in the tested GP samples was <0.035 mg/kg. PAHs have carcinogenic and mutagenic properties. Animals exposed to PAHs experience disorders of the immune system, urinary system, body fluids, and skin and lung damage. In studies of feed mixtures for pigs and cows, PAHs were detected at concentrations of 0.082 and 0.128 mg/kg [61], while in a study published by Zeko-Pivač et al. [5], they were <0.020 mg/kg LOQ for raw and fermented brewers spent grain. According to EU DIRECTIVE 2002/32/ EC [60], the maximum allowed concentration of aflatoxin in animal feed is 0.02 mg/kg.

#### 3.2.7. Content of Total Phenolic Compounds (TPC), Total Flavonoids (TF), Total Extractable Proanthocyanidins (TPA), and Antioxidant Activity of GP Extracts

**Figure 5:**
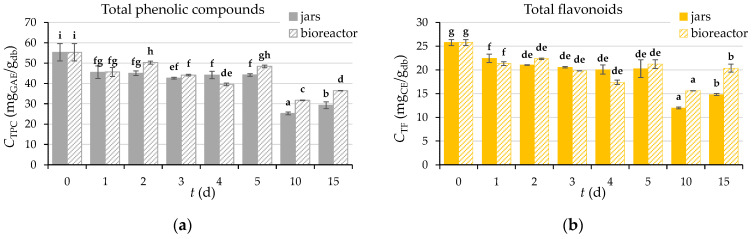

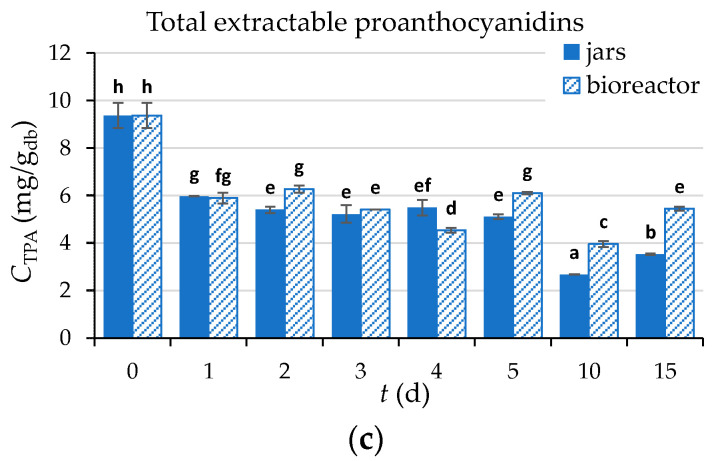
The content (*C*) of total phenolic compounds, TPC (**a**), total flavonoids, TF (**b**) and total extractable proanthocyanidins, TPA (**c**) in extracts obtained from GP before (day “0”) and after SSF with *R. oryzae* in laboratory jars and tray bioreactor (days 1–5, 10, 15). Samples marked with different lowercase letters of the alphabet are statistically significantly different from each other (*p* < 0.05, post-hoc Duncan’s multiple range test).

**Figure 6:**
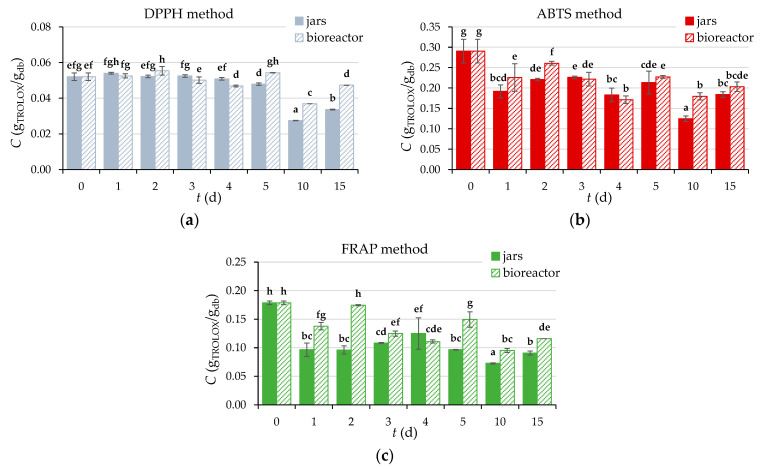
Antioxidant activity (DPPH method (**a**), ABTS method (**b**), FRAP method (**c**)) of extracts obtained from GP before (day “0”) and after SSF (days 1–5, 10, 15) using *R. oryzae* in laboratory jars and tray bioreactor. Samples marked with different lowercase letters of the alphabet are statistically significantly different from each other (*p* < 0.05, post-hoc Duncan multiple range test).

The contents of TPC, TF, and TPA were determined in the extract of the initial biologically untreated GP (day “0”) and in the extracts obtained from GP after biological treatment with *R. oryzae* (days 1–5, 10, 15) (Figure 5), and antioxidant activity was measured by DPPH, FRAP, and ABTS methods (Figure 6). SSF did not increase the yield of TPC, TF, and TPA, whose content decreased by 47%, 43%, and 62% in the laboratory jars, respectively, and by 34%, 21%, and 42% in the tray bioreactor after the 15th day of fermentation, respectively (Figure 5).

The reason for the reduction of TPC, TF, and TPA is the enzymatic decomposition and/or polymerization of phenolic compounds released by the growth of the fungus [62]. Authors Papadaki et al. [63] also observed a 74% reduction in TPC after a 20-day SSF of GP with *P. pulmonarius* in their study, as did Troncozo et al. [64] by 20.48% after a 90-day SSF of GP with *Peniophora albobadia*. Das et al. [65] used *R. oryzae* for bioprocessing apple pomace under SSF conditions and obtained an 81.5% increase in TPC content after 14 days of fermentation, indicating an effective biodegradation of polysaccharides (cellulose and hemicellulose) and lignin by *R. oryzae*. Based on these findings, it is clear that the different chemical composition of the substrate may significantly affect the growth of microorganisms and consequently, the degradation of polysaccharides and lignin, as well as the content of phenolic compounds.

A similar trend as TPC, TF, and TPA is followed by the antioxidant activity of the extracts during the biological treatment of GP with *R. oryzae* (Figure 6), the decrease of which is a consequence of the degradation of the bioactive compounds responsible for antioxidant potential [46].

To more precisely determine the antioxidant activity of the obtained extracts, three methods (DPPH, ABTS, and FRAP) based on different reaction mechanisms were used in this work. Biological treatment of GP with *R. oryzae* reduced antioxidant activity measured by DPPH, ABTS, and FRAP methods by 35%, 37%, and 49% in laboratory jars, respectively, and by 10%, 30%, and 35% in a tray bioreactor, respectively, after 15 days of SSF. Leite et al. [48] investigated the effects of using different microorganisms and substrates in SSF on TPC yield and antioxidant activity. Cultivation of *R. oryzae* on GP (a mixture of white and black GP) resulted in an increase in TPC yield and antioxidant activity, while the use of grape seeds as a substrate had the opposite effect and resulted in a decrease after seven days of fermentation.

#### 3.2.8. The Effect of Biological Treatment by *R. oryzae* on the Recovery of Individual Phenolic Compounds from Grape Pomace

In contrast to the results obtained for the total phenolic compounds (TPC, TF, TPA), the analysis of the individual phenolic compounds in the extracts showed a positive influence of SSF on their extractability. Of the 20 quantified phenolic compounds in the extracts of GP, SSF with *R. oryzae* had a positive influence on the recovery of 11 individual phenolic compounds. Their content before (day “0”, *C*_o_) and after SSF (*C*_i,max_.—the maximum content of an individual phenolic compound in the extract) are presented in Table 5. A graphic representation of the results of the dimensionless content of phenolic compounds before (“0” day) and after biological treatment of GP by *R. oryzae* for 1–5, 10, and 15 days is given in the Supplementary Materials in Figure S1.

The extractability of gallic acid, ellagic acid, *p*-hydroxybenzoic acid, syringic acid, vanillic acid and 3,4-dihydroxybenzoic acid, *p*-coumaric acid, caffeic acid, epicatechin gallate, quercetin, and resveratrol increased 1.1- to 2.4-fold after treatment in laboratory jars and 1.1- to 2.5-fold after treatment in a tray bioreactor. A statistically significant (*p* < 0.05) increase in the extractability of individual phenolic compounds from GP after biological treatment in laboratory jars was observed for all compounds listed in Table 5, except for resveratrol. After biological treatment in a tray bioreactor, a statistically significant (*p* < 0.05) increase in extractability was observed for all compounds listed in Table 5, except for vanillic acid and *p*-coumaric acid.

As can be seen from the results in Table 5, the process conducted in the tray bioreactor proved to be more effective in increasing the extraction yield of caffeic acid, epicatechin gallate, quercetin, and resveratrol (according to independent Student’s *t*-test, with a confidence level of 95%). Furthermore, *p*-hydroxybenzoic acid, syringic acid, and 3,4-dihydroxybenzoic acid showed a higher yield in the process carried out in the laboratory jars.

For ferulic acid, *o*-coumaric acid, catechin, epicatechin, gallocatechin gallate, rutin, kaempferol, procyanidin B1, and ε-viniferin, no increase in yield was observed after SSF. These results are presented graphically as dimensionless phenolic compound content before (“0” day) and after biological treatment (1–5, 10, and 15 days) are included in the Supplementary Materials as Figure S2.

The differential effect of SSF on the content of total and individual phenolic compounds is due to the fact that the Folin–Ciocalteu reagent used for the determination of TPC reacts not only with phenolic compounds, but also with amino acids, sugars, and other reducing substances in the extracts. Such components were present in higher concentrations in the extracts of GP before SSF than after SSF. This may lead to differences in the content of phenolic compounds in the extracts, which were determined by spectrophotometric and chromatographic methods. This observation was also made by Zambrano et al. [62] in their study investigating the influence of SSF and direct enzymatic treatment (using a cellulolytic cocktail) on the extractability of total and individual phenolic compounds from GP, apple pomace, and pitaya residues. Stratil et al. [66] described that ascorbic acid and mono- and disaccharides, such as glucose, fructose, and sucrose, which are present in fruits and vegetables, may cause interference in the Folin–Ciocalteu method. The final absorbance value when measured depends on the structure of the molecule (the reactivity of the individual phenolic hydroxyl) and is usually proportional to the number of reacting phenolic hydroxyl groups.

#### 3.2.9. Correlation between Hydrolytic and Lignolytic Enzymes and Individual Phenolic Compounds from Grape Pomace

During the SSF process, the activities of hydrolytic (*β*-glucosidase, xylanase, cellulase, invertase) and lignolytic enzymes (laccase, manganese peroxidase; MnP, lignin peroxidase; Lip) were measured to better understand their actions in catalyzing biotransformation processes during the biological processing of GP and their roles in increasing the extraction yield of phenolic compounds. Analysis of extracts obtained after biological treatment of GP with *R. oryzae* revealed the presence of all mentioned enzymes, which is presented in Table 6.

As shown in Table 6, a low activity of lignolytic enzymes (laccase, MnP, LiP) was observed during SSF with *R. oryzae*, although MnP activity was not detected on days 1 and 15 of SSF in the laboratory jars and on the first and fifth days in the tray bioreactor. The activity of the lignolytic enzymes increases with the duration of fermentation, and it can be assumed that the duration of SSF should be prolonged to obtain more significant activities of lignolytic enzymes and, consequently, better degradation of lignin and release of phenolic compounds from the lignin structure.

Of the hydrolytic enzymes studied, the strongest activity was observed for xylanase. It should be noted that efficient enzyme production during SSF depends on several factors, such as the specificity of the selected microorganism strain, the duration of the incubation period, and the incubation conditions. An important parameter affecting the SSF process and the enzyme production itself is the C/N ratio, which is usually between 15 and 35 in SSF processes [4,67,68]. In this study, the ratio TOC_E_:TN is quite high and increases during SSF and was in the range of 1:55–1:74 from the first to the 15th day of fermentation (note that this is the C/N ratio determined in GP extracts). To regulate the C/N ratio and the general nutrients in the substrate, it is preferable to mix GP with other lignocellulosic materials in the SSF process. For example, authors Teles et al. [46] used a mixture of wheat bran and GP for the cultivation of *A. niger* to produce hydrolytic enzymes and increase the yield of bioactive compounds. Filipe et al. [68] used a mixture of olive pomace and winery waste as a substrate for the cultivation of *A. niger* and *A. ibericus* to improve the production of cellulase and xylanase and the extractability of phenolic compounds. In a study by Díaz et al. [69], it was shown that the use of a mixture of GP and orange peel at a ratio of 1:1 (*w*/*w*) was effective for the production of hydrolytic enzymes in a tray bioreactor. The objective of this research was not the production of hydrolytic and lignolytic enzymes, but their activities were measured to determine correlation with recovered phenolic compounds.

In this work, the correlation between the above enzyme activities and the phenolic content in extracts from GP before (day “0”) and after SSF (days 1, 5, 10, and 15) with *R. oryzae* was evaluated. To evaluate the correlation between enzyme activity and certain groups of phenolic compounds, which include hydroxybenzoic acids (gallic acid, ellagic acid, *p*-hydroxybenzoic acid, syringic acid, vanillic acid, 3,4-dihydroxybenzoic acid), hydroxycinnamic acids (*o*-coumaric acid, *p*-coumaric acid, ferulic acid), flavan-3-ols (epicatechin gallate, gallocatechin gallate), flavonols (quercetin, kaempferol, rutin), procyanidins (procyanidin B1) and stilbenes (resveratrol, ε-viniferin), the sum of individual phenolic compounds from each group and the sum of all identified individual phenolic compounds (TP) were calculated to better represent the results. The correlation coefficient (*r*) indicates the linear relationship between the data, and its value can range from −1 (perfect negative linear relationship) to +1 (perfect positive linear relationship). The 0 value of the correlation coefficient indicates that there is no linear relationship between the data, and if its value is above +0.7 or below −0.7, this indicates a strong positive or negative linear relationship between the data. Other values indicate a medium or weak data correlation [70]. Table 7 and Table 8 show the correlation coefficient (*r*) between phenolic compound content (hydroxybenzoic acids; HBA, hydroxycinnamic acids; HCA, flavan-3-ols; F3L, flavonols; FL, procyanidin; PCD, stilbenes; ST, total phenolic compounds; TP) and the activity of hydrolytic (*β*-glucosidase, xylanase, cellulase, and invertase) and lignolytic (laccase, MnP, and LiP) enzymes in GP samples after SSF in laboratory jars and a tray bioreactor.

Table 7 shows that cellulase activity correlated with flavonoids (*r* = 0.717), whereas the other enzyme activities and phenolic compound groups did not correlate.

Table 8 shows that the correlation coefficient between the activity of hydrolytic enzymes (xylanase and invertase) and the activity of lignolytic enzymes with most polyphenolic compounds indicates a strong negative correlation, ranging from −0.700 to −0.994. The values of the other correlation coefficients indicate a moderate and weak relationship between the data.

Overall, an increase in the activity of the enzymes xylanase, laccase, MnP, and LiP resulted in lower contents of HBA, HCA, and F3L and a higher content of ST, where the correlation coefficient (*r* > 0.9) indicated a strong linear relationship.

From the results of the correlation between enzymes and phenolic substances, it can be concluded that the activity of the studied enzymes is not necessarily proportional to the increase in the yield of phenolic compounds from GP. Teles et al. [46] measured the enzyme activities of xylanase, carboxymethylcellulase, polygalacturonase, *β*-glucosidase, and tannase during SSF of GP and wheat bran by *Aspergillus niger* and showed that enzyme activity was not necessarily proportional to the release of bioactive compounds from GP.

## 4. Conclusions

SSF by filamentous fungi has developed significantly in recent decades, mainly due to the economic and environmental benefits in valorizing agroindustrial byproducts and producing valuable compounds with a wide range of applications. Nevertheless, issues of consideration, such as the impossibility of biomass separation, a good understanding of fungal evolution and metabolism, and scale-up limitations and consequent low industrial applications, will be crucial for the future development of this technology.

This research contributes to this development by providing insight into the recovery of bioactive compounds from GP. Biological treatment by *R. oryzae* had a positive effect on the recovery of 11 phenolic compounds from GP, making SSF a potential technology for the production of high-value products. *R. oryzae* transformed GP, resulting in a change in chemical composition. This study confirmed that *R. oryzae* produces hydrolytic and lignolytic enzymes during growth at GP. However, it can be assumed that the relatively low enzyme activities are due to the growth of *R. oryzae* on a substrate (GP) with a high C/N ratio, which should be regulated before starting SSF. Additionally, the duration of SSF should be prolonged to achieve higher enzyme activities and thus more effective degradation of lignocellulosic biomass. Biological treatment of GP with *R. oryzae* can improve the environmental impact of waste management in wineries. In addition, analysis of undesirable substances has shown that GP does not contain harmful substances and can potentially be used for the production of nutrient-enriched animal feed.

This area of research has great potential for expansion, as there is the possibility of using biologically treated GP and isolating certain phenolic compounds that can be used as functional ingredients in food or feed to improve consumer health, digestibility, and more. In this regard, one of the major challenges for the future is certainly to develop an SSF procedure without sample sterilization while ensuring the dominance of the selected microorganism.

## Figures and Tables

**Table 1:** Buffer for the preparation of extracts for the measurement of enzyme activity.

Enzyme	Buffer	pH
Xylanase	50 mM sodium citrate buffer	5.3
Cellulase	50 mM sodium citrate buffer	4.8
*β*-glucosidase	100 mM sodium acetate buffer	5.0
Invertase	100 mM sodium acetate buffer	4.5
Laccase	50 mM sodium malonate buffer	4.5
Manganese peroxidase (MnP)	50 mM sodium malonate buffer	4.5
Lignin peroxidase (LiP)	50 mM sodium tartrate buffer	3.0

**Table 2:** Chemical composition of GP before (day “0”) and after SSF (day 5, 10, 15) by *R. oryzae* in laboratory jars and tray bioreactor.

Compound	GP	SSF in Laboratory Jars			SSF in a Tray Bioreactor		
	Day “0”	Day 5	Day 10	Day 15	Day 5	Day 10	Day 15
Ash (%_db_)	8.65 ± 0.00 c	8.19 ± 0.11 bc	8.30 ± 0.18 bc	7.59 ± 0.73 a	7.84 ± 0.14 ab	8.13 ± 0.09 abc	7.95 ± 0.08 ab
Proteins (%_db_)	11.35 ± 0.09 e	9.09 ± 0.18 b	8.41 ± 0.10 a	9.91 ± 0.10 d	9.41 ± 0.00 c	9.56 ± 0.10 c	11.31 ± 0.10 e
Free fats (%_db_)	7.50 ± 0.20 a	9.01 ± 0.29 b	10.66 ± 0.29 d	10.77 ± 0.10 d	11.13 ± 0.47 d	11.00 ± 0.03 d	9.73 ± 0.04 c
TOC_GP_ (%_db_)	72.18 ± 0.56 a	73.03 ± 0.20 ab	73.77 ± 1.17 bc	74.75 ± 0.21 cd	74.20 ± 0.56 c	75.33 ± 0.12 d	80.14 ± 0.47 e
NDF (%_db_)	52.81 ± 1.10 a	57.60 ± 0.64 b	58.64 ± 1.12 b	56.69 ± 0.20 b	53.16 ± 1.13 a	58.78 ± 1.44 b	61.72 ± 1.84 c
ADF (%_db_)	44.26 ± 2.41 a	52.18 ± 0.77 bc	52.84 ± 0.66 c	50.17 ± 0.55 b	44.84 ± 1.77 a	49.66 ± 1.40 b	50.60 ± 0.94 bc
ADL/lignin (%_db_)	31.35 ± 1.49 a	36.59 ± 0.72 cd	37.97 ± 0.33 d	34.28 ± 0.57 c	30.81 ± 0.26 a	35.45 ± 0.14 bc	37.11 ± 1.01 d
Hemicellulose (%_db_)	8.55 ± 1.68 c	5.42 ± 0.30 a	5.80 ± 1.72 a	6.52 ± 0.76 ab	8.32 ± 0.64 bc	9.12 ± 1.11 c	11.64 ± 0.20 d
Cellulose (%_db_)	12.90 ± 1.70 a	15.58 ± 0.05 bc	14.87 ± 0.27 bc	15.89 ± 0.01 c	14.03 ± 1.58 ab	14.97 ± 0.57 bc	13.96 ± 0.55 ab
Sucrose (mg/g_db_)	12.04 ± 0.27 e	6.21 ± 0.03 d	4.77 ± 0.03 e	4.28 ± 0.02 b	2.88 ± 0.09 a	4.40 ± 0.03 b	4.88 ± 0.14 c
Glucose (mg/g_db_)	7.01 ± 0.29 e	1.63 ± 0.04 b	2.13 ± 0.07 c	0.78 ± 0.49 a	1.98 ± 0.04 bc	2.64 ± 0.11 d	2.14 ± 0.16 c
Fructose (mg/g_db_)	1.50 ± 0.12 d	0.21 ± 0.02 bc	0.07 ± 0.01 a	0.15 ± 0.01 ab	0.30 ± 0.01 c	0.18 ± 0.01 b	0.19 ± 0.07 b
RS (mg_GLUCOSE_/g_db_)	30.00 ± 0.76 g	21.93 ± 0.20 f	18.77 ± 0.19 d	13.96 ± 0.01 c	19.78 ± 0.41 e	13.08 ± 0.37 b	9.19 ± 0.41 a
TOC_E_ (mg/g_db_)	79.25 ± 7.15 d	49.65 ± 1.30 b	45.27 ± 1.13 b	30.54 ± 1.18 a	63.78 ± 1.40 c	44.90 ± 0.47 b	26.50 ± 0.20 a
TN (mg/g_db_)	1.45 ± 0.00 e	0.85 ± 0.02 d	0.72 ± 0.02 c	0.41 ± 0.04 a	0.90 ± 0.02 d	0.66 ± 0.03 b	0.36 ± 0.04 a

All results are given as mean (*n* = 3) ± SD. Values in the same row labelled with different lowercase letters of the alphabet are statistically significantly different from each other (*p* < 0.05, post-hoc Duncan multiple range test), samples belonging to the population with the lowest mean value of each component are labelled with the letter “a”. TOC_GP_—total organic carbon in GP, NDF—neutral detergent fibers, ADF—acid detergent fibers, ADL—acid detergent lignin, RS—reducing sugars, TOC_E_—total organic carbon in the extract, TN—total nitrogen.

**Table 3:** Minerals and heavy metals detected in grape pomace before (day “0”) and after 15 days of SSF by *R. oryzae* in tray bioreactor.

Element	Unit	Content	
		Day “0”	Day 15
Phosphorus (P)	%	0.30	0.28
Calcium (Ca)	mg/kg	4967	5030
Potassium (K)	mg/kg	22,130	11,219
Iron (Fe)	mg/kg	103	87
Lead (Pb)	mg/kg	0.064	0.060
Cadmium (Cd)	mg/kg	0.025	0.027
Arsenic (As)	mg/kg	0.024	0.018

**Table 4:** PAHs, PCBs, and mycotoxins analysis in grape pomace before (day “0”) and after 15 days of SSF by *R. oryzae* in tray bioreactor.

Component	Unit	Content	
		Day “0”	Day 15
Harmful Substances and Pollutants			
PAHs	mg/kg	<0.020	<0.020
PCBs	mg/kg	<0.035	<0.035
Mycotoxins *			
Aflatoxins	mg/kg	<0.003	<0.003
Fumonisins	mg/kg	<0.030	<0.030
Zearalenone	mg/kg	<0.030	<0.030
T-2/HT-2 toxin	µg/kg	<20	<20
Deoxynivalenol	mg/kg	<0.020	<0.020
Ochratoxin A	mg/kg	<0.001	<0.001

PAHs—polyaromatic hydrocarbons (benz(a)anthracene, benzo(a)pyrene, benzo(b)fluoranthene, and chrysene); PCB—polychlorinated biphenyls (polychlorinated biphenyls 28, 52, 101, 118, 135, 153, and 180); aflatoxins (aflatoxin B1, B2, G1, G2); fumonisins (fumonisin B1 i B2). * Accredited method in the flexible area according to HRN EN ISO/IEC 17025:2017.

**Table 5:** The content of individual phenolic compounds in extracts obtained from GP before SSF (day “0”, *C*_o_) and after SSF by *R. oryzae* in laboratory jars and in a tray bioreactor (maximum content of individual phenolic compounds in GP extracts recorded after a certain duration of SSF, *C*_i,max._).

Phenolic Compounds	Day “0”	SSF in Laboratory Jars			SSF in Tray Bioreactor			*p* ***
	*C*_o_ (µg/g_db_) *	*C*_i,max._ (µg/g_db_) *	*p* **	*t*_SSF_ (d)	*C*_i,max._ (µg/g_db_) *	*p* **	*t*_SSF_ (d)	
Phenolic acid (hydroxybenzoic acids)								
GA	307.20 ± 3.10	447.88 ± 33.15	0.0149	4.	490.77 ± 15.31	0.0033	4.	0.1117
EA	208.44 ± 4.42	235.50 ± 6.27	0.0483	3.	233.49 ± 1.62	0.0188	10.	0.6183
*p*-HBA	6.07 ± 0.44	14.30 ± 0.79	0.0006	4.	11.79 ± 0.17	0.0008	3.	0.0057
SA	236.63 ± 1.28	424.04 ± 4.96	0.0004	5.	282.00 ± 18.34	0.0441	1.	0.0002
VA	80.81 ± 1.31	97.49 ± 0.79	0.0053	2.	93.68 ± 6.57	0.0514	1.	0.3749
3,4-DHBA	111.51 ± 2.17	234.14 ± 9.27	0.0029	10.	194.24 ± 3.78	0.0017	4.	0.0023
Phenolic acid (hydroxycinnamic acids)								
*p*-CoA	8.76 ± 0.70	9.65 ± 0.55	0.0098	4.	10.23 ± 0.17	0.1000	4.	0.1544
CAF	13.12 ± 0.46	15.07 ± 0.17	0.0070	1.	17.14 ± 0.99	0.0058	1.	0.0235
Flavan-3-ol								
EPG	319.26 ± 3.39	366.73 ± 5.08	0.0004	1.	453.49 ± 15.62	0.0028	1.	0.0008
Flavonol								
QU	403.10 ± 9.04	657.79 ± 13.33	0.0001	15.	714.91 ± 15.92	0.0021	10.	0.0089
Stilbene								
RES	30.40 ± 0.69	30.63 ± 0.37	0.7370	3.	76.19 ± 0.41	0.0000	15.	0.0000

GA—gallic acid; EA—ellagic acid; *p*-HBA—*p*-hidroxybenzoic acid; SA—syringic acid; VA—vanillic acid; 3,4-DHBA—3,4-dihydroxybenzoic acid; *p*-CoA—*p*-coumaric acid; CAF—caffeic acid; EPG—epicatechin gallate; QU—quercetin; RES—resveratrol. * Results are expressed as mean (*n* = 3) ± SD. ** To compare the mean values of the concentration of individual phenolic compounds between day “0” (biologically untreated sample of GP) and the day of fermentation when the maximum yield of individual phenolic compounds was reached, the Student’s *t*-test was used for dependent samples with a confidence level of 95% (red colored values—statistically significant difference with *p* < 0.05). *** To compare the mean values of the concentration of individual phenolic compounds between GP samples biologically treated in laboratory jars and in the tray bioreactor when the maximum yield of individual phenolic compounds was reached, the Student’s *t*-test for independent samples with a confidence level of 95% was used (red colored values—statistically significant difference with *p* < 0.05).

**Table 6:** Hydrolytic and lignolytic enzyme activities during SSF (day 1, 5, 10, and 15) of GP by *R. oryzae* in laboratory jars and tray bioreactor.

*t*_SSF_ (d)	Hydrolytic Enzymes				Lignolytic Enzymes *		
	*β*-Glucosidase (U/g_db_)	Xylanase (U/g_db_)	Cellulase (U/g_db_)	Invertase (U/g_db_)	Laccase (U/g_db_)	MnP (U/g_db_)	LiP (U/g_db_)
Laboratory jars							
1.	0.421	25.517	0.234	n.d.	0.005	n.d.	0.013
5.	0.208	62.964	0.675	n.d.	0.001	0.001	0.006
10.	0.315	40.054	n.d.	8.198	0.001	0.005	0.039
15.	0.347	33.181	1.020	6.316	0.002	n.d.	0.040
Tray bioreactor							
1.	0.290	9.271	0.293	6.135	0.005	n.d.	0.036
5.	0.533	17.684	0.685	2.700	0.009	n.d.	0.026
10.	0.440	46.133	0.666	10.706	0.071	0.018	0.069
15.	0.430	47.673	0.523	7.344	0.072	0.018	0.074

* manganese peroxidase (MnP), lignin peroxidase (LiP). n.d.—enzyme activity was not detected.

**Table 7:** Correlation coefficient (*r*) between the concentration of certain groups of phenolic compounds and the activity of hydrolytic and lignolytic enzymes in GP after SSF with *R. oryzae* in laboratory jars.

Phenolic Compounds *	Hidrolytic Enzymes				Lignolytic Enzymes **		
	*β*-Glucosidase	Xylanase	Cellulase	Invertase	Laccase	MnP	LiP
HBA	−0.433	0.555	−0.565	−0.552	−0.233	0.188	−0.739
HCA	0.149	0.014	−0.579	−0.755	0.404	−0.181	−0.790
F3L	0.116	0.051	−0.095	−0.970	0.474	−0.643	−0.919
FL	0.192	−0.323	0.717	0.533	0.014	−0.205	0.687
PCD	−0.476	0.618	−0.225	−0.810	−0.163	−0.201	−0.937
ST	−0.105	0.273	−0.274	−0.923	0.236	−0.398	−0.962
TP	−0.331	0.487	−0.154	−0.913	0.019	−0.379	−0.987

* hydroxybenzoic acids (HBA), hydroxycinnamic acids (HCA), flavan-3-ols (F3L), flavonols (FL), procyanidins (PCD), stilbenes (ST), total phenolic compounds (TP). ** manganese peroxidase (MnP), lignin peroxidase (LiP). Orange values—a strong negative correlation. Blue values—a strong positive correlation.

**Table 8:** Correlation coefficient (*r*) between the concentration of certain groups of phenolic compounds and the activity of hydrolytic and lignolytic enzymes in GP after SSF with *R. oryzae* in tray bioreactor.

Phenolic Compounds *	Hidrolytic Enzymes				Lignolytic Enzymes **		
	*β*-Glucosidase	Xylanase	Cellulase	Invertase	Laccase	MnP	LiP
HBA	−0.494	−0.854	−0.472	−0.257	−0.801	−0.790	−0.735
HCA	−0.349	−0.966	−0.431	−0.534	−0.943	−0.937	−0.897
F3L	−0.235	−0.994	−0.387	−0.700	−0.992	−0.989	−0.961
FL	0.162	0.240	0.477	0.570	0.253	0.238	0.161
PCD	0.638	−0.532	0.472	−0.781	−0.635	−0.669	−0.790
ST	0.203	0.917	0.253	0.508	0.908	0.910	0.903
TP	0.217	−0.792	0.133	−0.643	−0.838	−0.857	−0.922

* hydroxybenzoic acids (HBA), hydroxycinnamic acids (HCA), flavan-3-ols (F3L), flavonols (FL), procyanidins (PCD), stilbenes (ST), total phenolic compounds (TP). ** manganese peroxidase (MnP), lignin peroxidase (LiP). Orange values—a strong negative correlation. Blue values—a strong positive correlation.

## Data Availability

The data presented in this study are available upon request from the corresponding author.

## Supplementary Materials

The following supporting information can be downloaded at: https://www.mdpi.com/article/10.3390/microorganisms11040956/s1, Figure S1: Dimensionless content (*C* = *C*_i_/*C*_o_) of hydroxybenzoic acids (a–f), hydroxycinnamic acids (g–h), flavan-3-ol (i), flavonol (j), and stilbene (k) in GP extracts before biological treatment (“0“ day) and after 1–5, 10, and 15 days of biological treatment with *R. oryzae*; Figure S2: Dimensionless content (*C* = *C*_i_/*C*_o_) hydroxycinnamic acids (a–b), flavan-3-ols (c–e), flavonol (f–g), procyanidin (h), and stilbene (i) in GP extracts before biological treatment (“0“ day) and after 1–5, 10, and 15 days of biological treatment with *R. oryzae*.

## References

[1] Amaya-Chantaca D., Flores-Gallegos A.C., Iliná A., Aguilar C.N., Sepúlveda-Torre L., Ascacio-Vadlés J.A., Chávez-González M.L. (2022). Comparative Extraction Study of Grape Pomace Bioactive Compounds by Submerged and Solid-State Fermentation. J. Chem. Technol. Biotechnol..

[2] Cabezudo I., Galetto C.S., Romanini D., Furlán R.L.E., Meini M.R. (2022). Production of Gallic Acid and Relevant Enzymes by *Aspergillus niger* and *Aspergillus oryzae* in Solid-State Fermentation of Soybean Hull and Grape Pomace. Biomass Convers. Biorefin..

[3] Filippi K., Georgaka N., Alexandri M., Papapostolou H., Koutinas A. (2021). Valorisation of Grape Stalks and Pomace for the Production of Bio-Based Succinic Acid by *Actinobacillus succinogenes*. Ind. Crops Prod..

[4] Melanouri E.-M. Cultivating Pleurotus Ostreatus and Pleurotus Eryngii Mushroom Strains on Agro-Industrial Residues in Solid-State Fermentation. Part I: Screening for Growth, Endoglucanase, Laccase and Biomass Production in the Colonization Phase | Elsevier Enhanced Reader. https://reader.elsevier.com/reader/sd/pii/S2588913321000508?token=845D3C3D4D7E170E9F5CFC90D2EF513447FC3791C1ADA17C0DFBE775F1C4B0E0C30D016C2246A28A492F2A0DECD9CB1C&originRegion=eu-west-1&originCreation=20230227211512.

[5] Zeko-Pivač A., Bošnjaković A., Planinić M., Parlov Vuković J., Novak P., Jednačak T., Tišma M. (2022). Improvement of the Nutraceutical Profile of Brewer’s Spent Grain after Treatment with *Trametes versicolor*. Microorganisms.

[6] Meini M.-R., Cabezudo I., Galetto C.S., Romanini D. (2021). Production of Grape Pomace Extracts with Enhanced Antioxidant and Prebiotic Activities through Solid-State Fermentation by *Aspergillus niger* and *Aspergillus oryzae*. Food Biosci..

[7] Belenioti M., Mathioudaki E., Spyridaki E., Ghanotakis D., Chaniotakis N. (2023). Biodegradation of Phenolic Compounds from Grape Pomace of *Vitis vinifera* Asyrtiko by *Chlamydomonas reinhardtii*. J. Chem. Technol. Biotechnol..

[8] Farru G., Cappai G., Carucci A., De Gioannis G., Asunis F., Milia S., Muntoni A., Perra M., Serpe A. (2022). A Cascade Biorefinery for Grape Marc: Recovery of Materials and Energy through Thermochemical and Biochemical Processes. Sci. Total Environ..

[9] Tišma M., Bucić-Kojić A., Planinić M. (2021). Bio-Based Products from Lignocellulosic Waste Biomass: A State of the Art. Chem. Biochem. Eng. Q..

[10] Costa-Silva V., Anunciação M., Andrade E., Fernandes L., Costa A., Fraga I., Barros A., Marques G., Ferreira L., Rodrigues M. (2022). Biovalorization of Grape Stalks as Animal Feed by Solid State Fermentation Using White-Rot Fungi. Appl. Sci..

[11] Arancibia-Díaz A., Astudillo-Castro C., Altamirano C., Soto-Maldonado C., Vergara-Castro M., Córdova A., Zúñiga-Hansen M.E. (2023). Development of Solid-State Fermentation Process of Spent Coffee Grounds for the Differentiated Obtaining of Chlorogenic, Quinic, and Caffeic Acids. J. Sci. Food Agric..

[12] Paz-Arteaga S.L., Ascacio-Valdés J.A., Aguilar C.N., Cadena-Chamorro E., Serna-Cock L., Aguilar-González M.A., Ramírez-Guzmán N., Torres-León C. (2023). Bioprocessing of Pineapple Waste for Sustainable Production of Bioactive Compounds Using Solid-State Fermentation. Innov. Food Sci. Emerg. Technol..

[13] Li Q., Yi P., Zhang J., Shan Y., Lin Y., Wu M., Wang K., Tian G., Li J., Zhu T. (2023). Bioconversion of Food Waste to Crayfish Feed Using Solid-State Fermentation with Yeast. Environ. Sci. Pollut. Res..

[14] Pérez-Rodríguez N., Oliveira F., Pérez-Bibbins B., Belo I., Torrado Agrasar A., Domínguez J.M. (2014). Optimization of Xylanase Production by Filamentous Fungi in Solid-State Fermentation and Scale-up to Horizontal Tube Bioreactor. Appl. Biochem. Biotechnol..

[15] Londoño-Hernández L., Ramírez-Toro C., Ruiz H.A., Ascacio-Valdés J.A., Aguilar-Gonzalez M.A., Rodríguez-Herrera R., Aguilar C.N. (2017). Rhizopus Oryzae—Ancient Microbial Resource with Importance in Modern Food Industry. Int. J. Food Microbiol..

[16] Yafetto L., Odamtten G.T., Wiafe-Kwagyan M. (2023). Valorization of Agro-Industrial Wastes into Animal Feed through Microbial Fermentation: A Review of the Global and Ghanaian Case. Heliyon.

[17] Ma X., Gao M., Yin Z., Zhu W., Liu S., Wang Q. (2020). Lactic Acid and Animal Feeds Production from Sophora Flavescens Residues by *Rhizopus oryzae* Fermentation. Process Biochem..

[18] Hermansyah H., Andikoputro M.I., Alatas A. Production of Lipase Enzyme from *Rhizopus oryzae* by Solid State Fermentation and Submerged Fermentation Using Wheat Bran as Substrate. Proceedings of the AIP Conference.

[19] AOAC (1995). Official Method 942.05. Official Methods of Analysis of AOAC INTERNATIONAL.

[20] Goering H.K., Soest P.J.V. (1970). Forage Fiber Analyses (Apparatus, Reagents, Procedures, and Some Applications).

[21] AOAC (2005). Official Method 2001.11. Official Methods of Analysis of AOAC INTERNATIONAL.

[22] AOAC (2005). Official Method 945.16. Official Methods of Analysis of AOAC INTERNATIONAL.

[23] Barreira J.C.M., Oliveira M.B.P.P., Ferreira I.C.F.R. (2014). Development of a Novel Methodology for the Analysis of Ergosterol in Mushrooms. Food Anal. Methods.

[24] Waterhouse A.L., Wrolstad R.E. (2001). Determination of Total Phenolics. Current Protocols in Food Analytical Chemistry.

[25] Marinova D., Ribarova F., Atanassova M. (2005). Total Phenolics and Total Flavonoids in Bulgarian Fruits and Vegetables. J. Univ. Chem. Technol. Metall..

[26] Škerget M., Kotnik P., Hadolin M., Hraš A.R., Simonič M., Knez Ž. (2005). Phenols, Proanthocyanidins, Flavones and Flavonols in Some Plant Materials and Their Antioxidant Activities. Food Chem..

[27] Bucić-Kojić A., Šelo G., Zelić B., Planinić M., Tišma M. (2017). Recovery of Phenolic Acid and Enzyme Production from Corn Silage Biologically Treated by Trametes Versicolor. Appl. Biochem. Biotechnol..

[28] Šelo G., Planinić M., Tišma M., Grgić J., Perković G., Koceva Komlenić D., Bucić-Kojić A. (2022). A Comparative Study of the Influence of Various Fungal-Based Pretreatments of Grape Pomace on Phenolic Compounds Recovery. Foods.

[29] Alarcón E., Hernández C., García G., Ziarelli F., Gutiérrez-Rivera B., Musule R., Vázquez-Marrufo G., Gardner T.G. (2021). Changes in Chemical and Structural Composition of Sugarcane Bagasse Caused by Alkaline Pretreatments [Ca(OH)_2_ and NaOH] Modify the Amount of Endoglucanase and *β*-Glucosidase Produced by *Aspergillus niger* in Solid-State Fermentation. Chem. Eng. Commun..

[30] Miller G.L. (1959). Use of Dinitrosalicylic Acid Reagent for Determination of Reducing Sugar. Anal. Chem..

[31] Croatian Standards Institute HRN ISO 6491:2001. http://31.45.242.218/HZN/todb.nsf/wFrameset2?OpenFrameSet&Frame=Down&Src=%2FHZN%2Ftodb.nsf%2FNormaSve%2Fc1256c8f003565d5c1256d29003e1e06%3FOpenDocument%26AutoFramed.

[32] Croatian Standards Institute HRN EN ISO/IEC 17025:2017. http://31.45.242.218/HZN/todb.nsf/wFrameset2?OpenFrameSet&Frame=Down&Src=%2FHZN%2Ftodb.nsf%2FNormaSve%2Fda093fd23afffcb8c12580a4003bbaf4%3FOpenDocument%26AutoFramed.

[33] Bucić-Kojić A., Planinić M., Tomas S., Jakobek L., Šeruga M. (2009). Influence of Solvent and Temperature on Extraction of Phenolic Compounds from Grape Seed, Antioxidant Activity and Colour of Extract. Int. J. Food Sci. Technol..

[34] Re R., Pellegrini N., Proteggente A., Pannala A., Yang M., Rice-Evans C. (1999). Antioxidant Activity Applying an Improved ABTS Radical Cation Decolorization Assay. Free Radical Biol. Med..

[35] Benzie I.F.F., Strain J.J. (1996). The Ferric Reducing Ability of Plasma (FRAP) as a Measure of “Antioxidant Power”: The FRAP Assay. Anal. Biochem..

[36] Bailey M.J., Biely P., Poutanen K. (1992). Interlaboratory Testing of Methods for Assay of Xylanase Activity. J. Biotechnol..

[37] Ghose T.K. (1987). Measurement of Cellulase Activities. Pure Appl. Chem..

[38] Adney B., Baker J. (2008). Measurement of Cellulase Activities: Laboratory Analytical Procedure (LAP). Technical Report NREL/TP-510-42628. https://www.nrel.gov/docs/gen/fy08/42628.pdf.

[39] Karpe A.V., Dhamale V.V., Morrison P.D., Beale D.J., Harding I.H., Palombo E.A. (2017). Winery Biomass Waste Degradation by Sequential Sonication and Mixed Fungal Enzyme Treatments. Fungal Genet. Biol..

[40] Margetić A., Vujčić Z. (2017). Comparative Study of Stability of Soluble and Cell Wall Invertase from *Saccharomyces cerevisiae*. Prep. Biochem. Biotechnol..

[41] Lueangjaroenkit P., Kunitake E., Sakka M., Kimura T., Teerapatsakul C., Sakka K., Chitradon L. (2020). Light Regulation of Two New Manganese Peroxidase-Encoding Genes in *Trametes polyzona* KU-RNW027. Microorganisms.

[42] Field J.A., Vledder R.H., van Zelst J.G., Rulkens W.H. (1996). The Tolerance of Lignin Peroxidase and Manganese-Dependent Peroxidase to Miscible Solvents and the in Vitro Oxidation of Anthracene in Solvent: Water Mixtures. Enzyme Microb. Technol..

[43] Linko S., Haapala R. (1993). A Critical Study of Lignin Peroxidase Activity Assay by Veratryl Alcohol Oxidation. Biotechnol. Tech..

[44] Van Kuijk S.J.A., Sonnenberg A.S.M., Baars J.J.P., Hendriks W.H., Cone J.W. (2015). Fungal Treatment of Lignocellulosic Biomass: Importance of Fungal Species, Colonization and Time on Chemical Composition and in Vitro Rumen Degradability. Anim. Feed. Sci. Technol..

[45] Šelo G., Planinić M., Tišma M., Tomas S., Koceva Komlenić D., Bucić-Kojić A. (2021). A Comprehensive Review on Valorization of Agro-Food Industrial Residues by Solid-State Fermentation. Foods.

[46] Teles A.S.C., Chávez D.W.H., Oliveira R.A., Bon E.P.S., Terzi S.C., Souza E.F., Gottschalk L.M.F., Tonon R.V. (2019). Use of Grape Pomace for the Production of Hydrolytic Enzymes by Solid-State Fermentation and Recovery of Its Bioactive Compounds. Food Res. Int..

[47] Niu D., Zuo S., Jiang D., Tian P., Zheng M., Xu C. (2018). Treatment Using White Rot Fungi Changed the Chemical Composition of Wheat Straw and Enhanced Digestion by Rumen Microbiota In Vitro. Anim. Feed Sci. Technol..

[48] Leite P., Silva C., Salgado J.M., Belo I. (2019). Simultaneous Production of Lignocellulolytic Enzymes and Extraction of Antioxidant Compounds by Solid-State Fermentation of Agro-Industrial Wastes. Ind. Crops Prod..

[49] Mushollaeni W., Tantalu L. (2020). Anthocyanin and Nutritional Contents of Fermented Lebui Bean (*Cajanus* sp.) through SSF Method and Induced by *Rhizopus* sp. and *Saccharomyces* sp.. IOP Conf. Ser. Earth Environ. Sci.

[50] Singh S., Khajuria R. (2019). Regulation by Metal Ions. New and Future Developments in Microbial Biotechnology and Bioengineering: Microbial Secondary Metabolites Biochemistry and Applications.

[51] Bastos R.G., Ribeiro H.C. (2020). Citric Acid Production by the Solid-State Cultivation Consortium of and from Sugarcane Bagasse. Open Biotechnol. J..

[52] Yu H., Xie B., Khan R., Dong J., Shen G. (2021). The Changes in Macronutrients and Microbial Community Structure during the Co-Composting of White Wine Distillers’ Grains and Potassium Silicate. J. Clean. Prod..

[53] Dulf F.V., Vodnar D.C., Toşa M.I., Dulf E.-H. (2020). Simultaneous Enrichment of Grape Pomace with γ-Linolenic Acid and Carotenoids by Solid-State Fermentation with *Zygomycetes fungi* and Antioxidant Potential of the Bioprocessed Substrates. Food Chem..

[54] Suleiman W., El-Sheikh H., Abu-Elreesh G., Hashem A. (2018). Isolation and Screening of Promising Oleaginous *Rhizopus* sp. and Designing of Taguchi Method for Increasing Lipid Production. J. Innov. Pharm. Biol. Sci..

[55] KKupski L., Cipolatti E., Rocha M.D., Oliveira M.D.S., Souza-Soares L.D.A., Badiale-Furlong E. (2012). Solid-State Fermentation for the Enrichment and Extraction of Proteins and Antioxidant Compounds in Rice Bran by *Rhizopus oryzae*. Braz. Arch. Biol. Technol..

[56] Larios-Cruz R., Buenrostro-Figueroa J., Prado-Barragán A., Rodríguez-Jasso R.M., Rodríguez-Herrera R., Montañez J.C., Aguilar C.N. (2019). Valorization of Grapefruit By-Products as Solid Support for Solid-State Fermentation to Produce Antioxidant Bioactive Extracts. Waste Biomass Valoriz..

[57] Kumar V., Ahluwalia V., Saran S., Kumar J., Patel A.K., Singhania R.R. (2021). Recent Developments on Solid-State Fermentation for Production of Microbial Secondary Metabolites: Challenges and Solutions. Bioresour. Technol..

[58] Botella C., Diaz A., de Ory I., Webb C., Blandino A. (2007). Xylanase and Pectinase Production by *Aspergillus awamori* on Grape Pomace in Solid State Fermentation. Process Biochem..

[59] Lopes V.R.O., Farias M.A., Belo I.M.P., Coelho M.A.Z. (2016). Nitrogen Sources on TPOMW Valorization through Solid State Fermentation Performed by *Yarrowia lipolytica*. Braz. J. Chem. Eng..

[60] Eur-LEX Directive 2002/32/EC of the European Parliament and of the Council of 7 May 2002 on Undesirable Substances in Animal Feed. https://eur-lex.europa.eu/eli/dir/2002/32/2019-11-28.

[61] Şahin T., Dalğa S., Ölmez M., Şahin T., Dalğa S., Ölmez M. (2022). Polycyclic Aromatic Hydrocarbons (PAHs) and Their Importance in Animal Nutrition.

[62] Zambrano C., Kotogán A., Bencsik O., Papp T., Vágvölgyi C., Mondal K.C., Krisch J., Takó M. (2018). Mobilization of Phenolic Antioxidants from Grape, Apple and Pitahaya Residues via Solid State Fungal Fermentation and Carbohydrase Treatment. LWT.

[63] Papadaki A., Kachrimanidou V., Papanikolaou S., Philippoussis A., Diamantopoulou P. (2019). Upgrading Grape Pomace through *Pleurotus* spp. Cultivation for the Production of Enzymes and Fruiting Bodies. Microorganisms.

[64] Troncozo M.I., Figoli C.B., Franco M.E.E., Mirífico M.V., Bosch A., Rajchenberg M., Balatti P.A., Saparrat M.C.N. (2020). Biotransformation of Grape Pomace from *Vitis labrusca* by *Peniophora albobadia* LPSC # 285 (Basidiomycota). An. Acad. Bras. Ciênc..

[65] Das R.K., Brar S.K., Verma M. (2015). A Fermentative Approach towards Optimizing Directed Biosynthesis of Fumaric Acid by *Rhizopus oryzae* 1526 Utilizing Apple Industry Waste Biomass. Fungal Biol..

[66] Stratil P., Klejdus B., Kubáň V. (2006). Determination of Total Content of Phenolic Compounds and Their Antioxidant Activity in VegetablesEvaluation of Spectrophotometric Methods. J. Agric. Food Chem..

[67] Ferreira J.A., Lennartsson P.R., Edebo L., Taherzadeh M.J. (2013). Zygomycetes-Based Biorefinery: Present Status and Future Prospects. Bioresour. Technol..

[68] Filipe D., Fernandes H., Castro C., Peres H., Oliva-Teles A., Belo I., Salgado J.M. (2020). Improved Lignocellulolytic Enzyme Production and Antioxidant Extraction Using Solid-state Fermentation of Olive Pomace Mixed with Winery Waste. Biofuels Bioprod. Bioref..

[69] Díaz A.B., Alvarado O., de Ory I., Caro I., Blandino A. (2013). Valorization of Grape Pomace and Orange Peels: Improved Production of Hydrolytic Enzymes for the Clarification of Orange Juice. Food Bioprod. Process..

[70] Ratner B. (2009). The Correlation Coefficient: Its Values Range between +1/−1, or Do They?. J. Target Meas. Anal. Mark..

