# Supplementary material for: Bioconversion of Grape Pomace with Rhizopus oryzae under Solid-State Conditions: Changes in the Chemical Composition and Profile of Phenolic Compounds
Source: Microorganisms. 2023 Apr 6;11(4):956. doi: 10.3390/microorganisms11040956 (PMC10143194; doi:10.3390/microorganisms11040956)
Supplement: Supplementary file 1 [file microorganisms-11-00956-s001.zip › microorganisms-2301704-supplementary.pdf]

Hydroxybenzoic acids

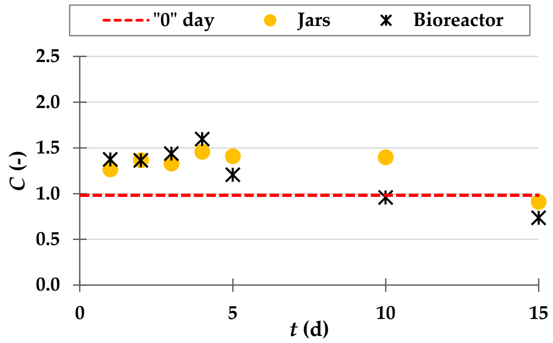

(**a**) Gallic acid ($C_{\text{“0” day}}$ = 307.21 ± 3.10 μg/g_{db})

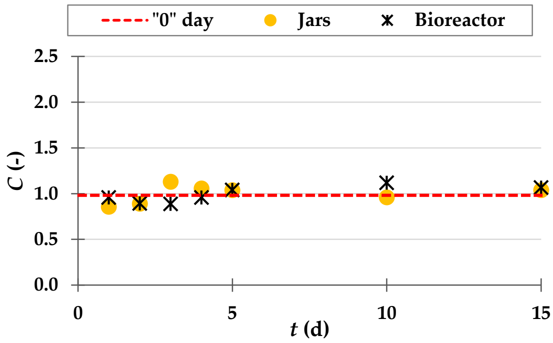

(**b**) Ellagic acid ($C_{\text{“0” day}}$ = 208.44 ± 4.42 μg/g_{db})

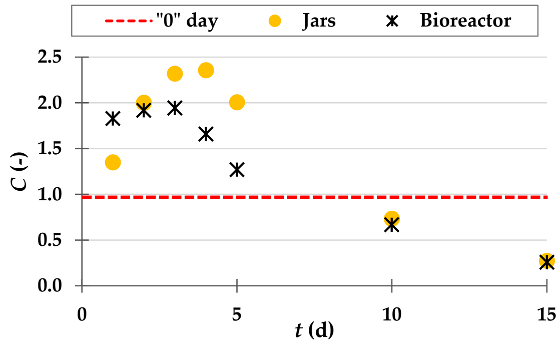

(**c**) *p*-Hydroxybenzoic acid ($C_{\text{“0” day}}$ = 6.07 ± 0.44 μg/g_{db})

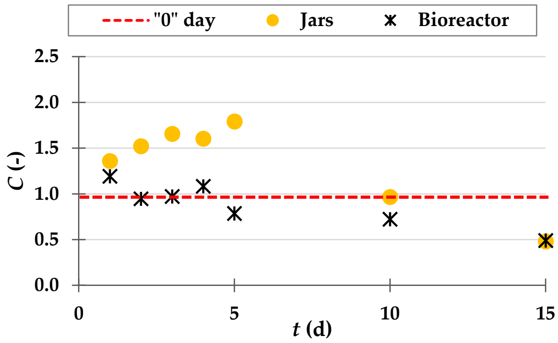

(**d**) Syringic acid ($C_{\text{“0” day}}$ = 236.63 ± 1.28 μg/g_{db})

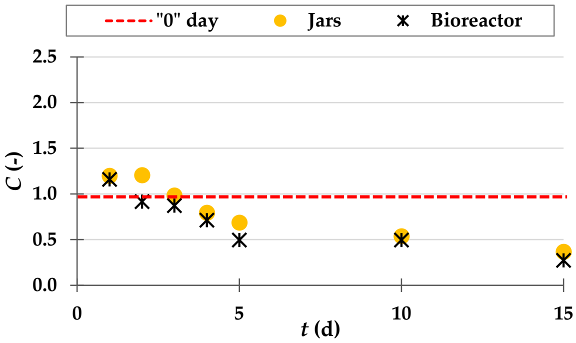

(**e**) Vanillic acid ($C_{\text{“0” day}}$ = 80.81 ± 1.31 μg/g_{db})

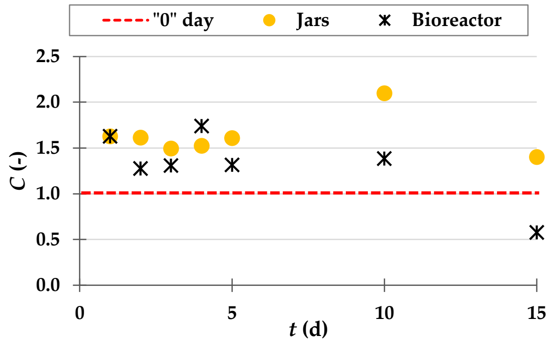

(**f**) 3.4-Dihydroxybenzoic acid ($C_{\text{“0” day}}$ = 111.51 ± 2.17 μg/g_{db})

Hydroxycinnamic acids

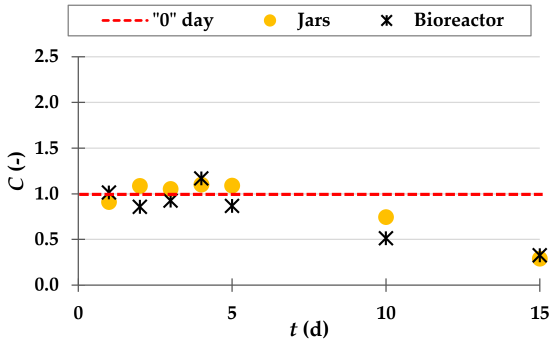

(**g**) *p*-Coumaric acid ($C_{\text{“0” day}}$ = 8.76 ± 0.70 μg/g_{db})

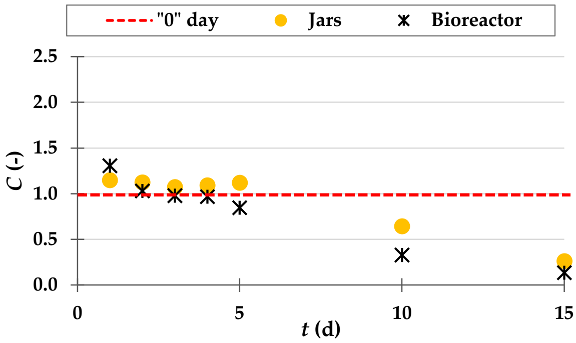

(**h**) Caffeic acid ($C_{\text{“0” day}}$ = 13.12 ± 0.46 μg/g_{db})

Flavan-3-ol

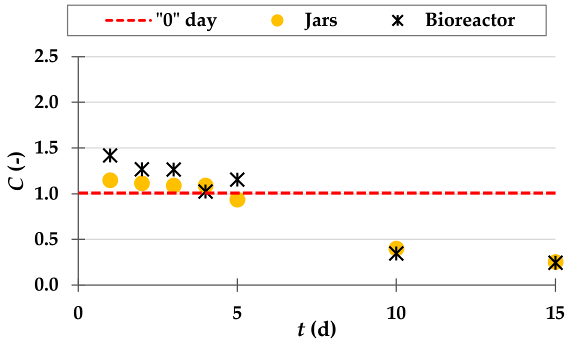

(**i**) Epicatechin gallate ($C_{\text{“0” day}}$ = 319.26 ± 3.39 μg/g_{db})

Flavonol

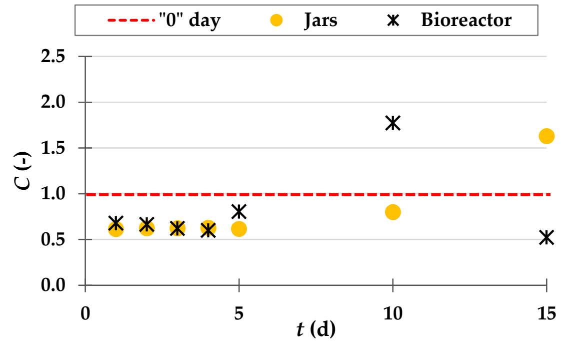

(**j**) Quercetin ($C_{\text{“0” day}}$ = 403.10 ± 9.04 μg/g_{db})

Stilbene

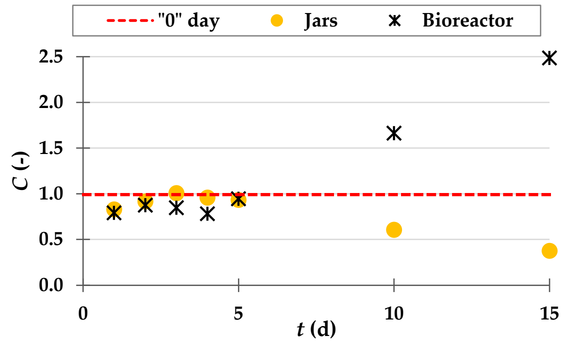

(**k**) Resveratrol ($C_{\text{“0” day}}$ = 30.40 ± 0.69 μg/g_{db})

Figure S1 Dimensionless content ($C = C_i/C_o$) of hydroxybenzoic acids (a-f), hydroxycinnamic acids (g-h), flavan-3-ol (i), flavonol (j), and stilbene (k) in GP extracts before biological treatment (“0“ day) and after 1-5, 10 and 15 days of biological treatment with *R. oryzae*.

Hydroxycinnamic acids

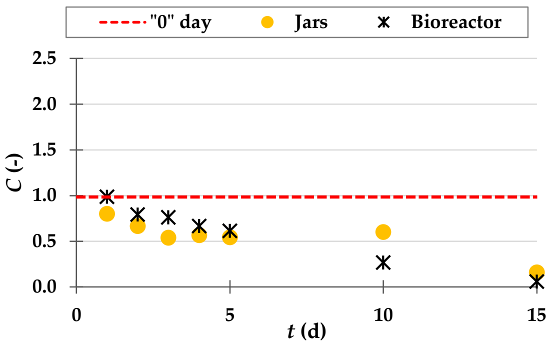

(**a**) Ferulic acid ($C_{\text{“0” day}}$ = 5.70 ± 0.25 μg/g_{db})

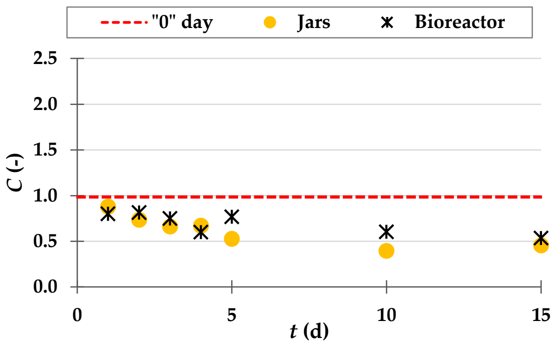

(**b**) *o*-Coumaric acid ($C_{\text{“0” day}}$ = 13.82 ± 0.27 μg/g_{db})

Flavan-3-ols

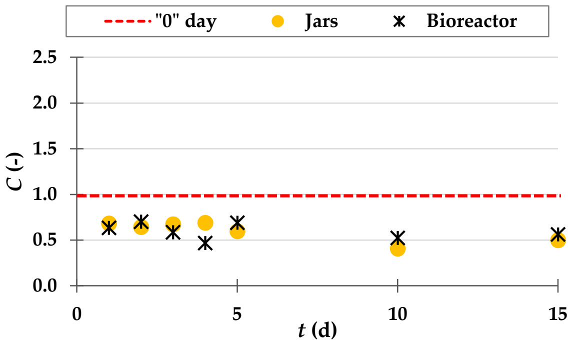

(**c**) Catechin ($C_{\text{“0” day}}$ = 2285.79 ± 31.83 μg/g_{db})

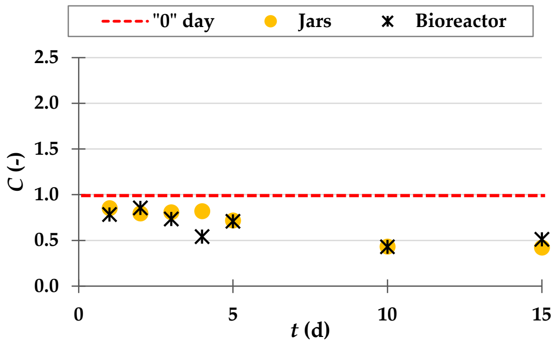

(**d**) Epicatechin ($C_{\text{“0” day}}$ = 1303.71 ± 5.00 μg/g_{db})

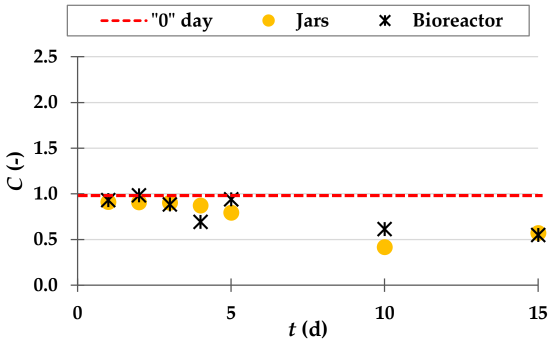

(**e**) Gallocatechin gallate ($C_{\text{“0” day}}$ = 605.28 ± 16.50 μg/g_{db})

Flavonol

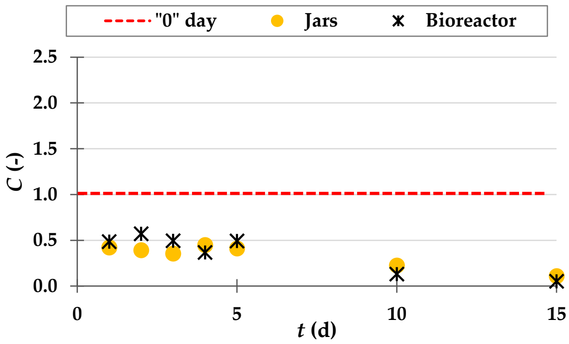

(**f**) Rutin ($C_{\text{“0” day}}$ = 160.29 ± 6.32 μg/g_{db})

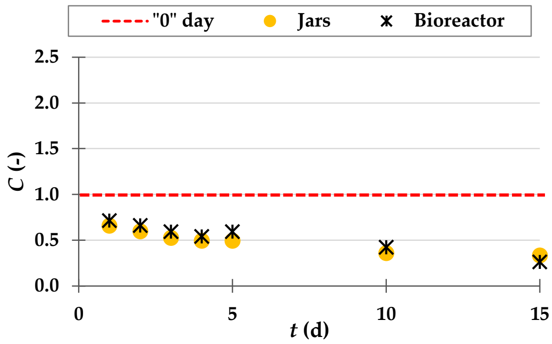

(**g**) Kaempferol ($C_{\text{“0” day}}$ = 19.80 ± 0.06 μg/g_{db})

Procyanidin

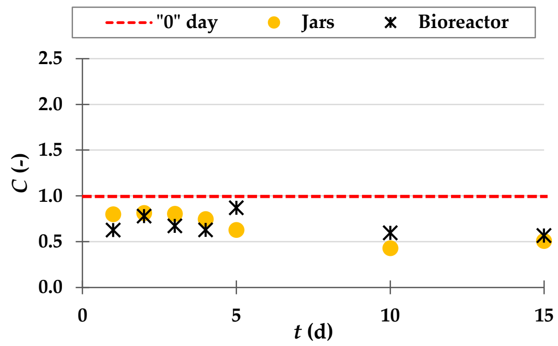

(**h**) Procyanidin B1 ($C_{\text{“0” day}}$ = 1235.22 ± 31.52 µg/g_{db})

Stilbene

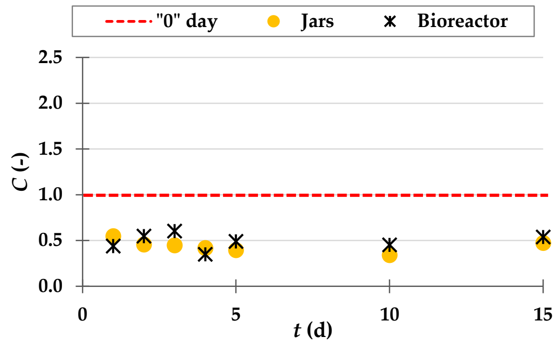

(**i**) ε-Viniferin ($C_{\text{“0” day}}$ = 319.26 ± 3.39 µg/g_{db})

Figure S2 Dimensionless content ($C = C_i/C_o$) hydroxycinnamic acids (a-b), flavan-3-ols (c-e), flavonol (f-g), procyanidin (h) and stilbene (i) in GP extracts before biological treatment (“0“ day) and after 1-5, 10 and 15 days of biological treatment with *R. oryzae*.
